# Molecular arrangement and modulation of extrasynaptic αβδ GABA_A_ receptors

**DOI:** 10.1038/s41467-026-76879-5

**Published:** 2026-08-18

**Authors:** Stephanie A. Nestorow, Wan-Na Chen, Daniel Bertrand, Ayla A. Wahid, Caroline E. Wyatt, Martin Mortensen, Thomas Clairfeuille, Michael Reutlinger, Gregoire Friz, Els Pardon, Jan Steyaert, Graham Ladds, Giuseppe Cecere, Trevor G. Smart, Maria-Clemencia Hernandez, Lisa Joedicke, Paul S. Miller

**Affiliations:** 1 Department of Pharmacology, University of Cambridge, Cambridge, UK; 2 HiQScreen Sàrl, 6, rte de Compois, 1222 Vésenaz, Geneva, Switzerland; 3 Department of Neuroscience, Physiology and Pharmacology, University College London, London, UK; 4 Roche Pharma Research and Early Development, Therapeutic Modalities, Roche Innovation Center Basel, Basel, Switzerland; 5 Roche Pharma Research and Early Development, Neuroscience and Rare Diseases, Roche Innovation Center Basel, Basel, Switzerland; 6 Structural Biology Brussels, Vrije Universiteit Brussel (VUB), Brussels, Belgium; 7 VIB-VUB Center for Structural Biology, VIB, Brussels, Belgium

**Keywords:** Cryoelectron microscopy, Molecular neuroscience, Ligand-gated ion channels, Permeation and transport

## Abstract

γ-aminobutyric acid Type-A (GABA_A_) αβδ receptors regulate neuronal excitability and contribute to sleep, mood and motor coordination. However, the molecular arrangements of these receptors and the basis of GABA activation and δ-selective drug modulation remain unclear. We solve cryo-EM structures of α4β3δ receptors in an α-β-α-β-δ arrangement that contains two GABA binding pockets. GABA binding supports a classical β-subunit tilt and an outward configuration of the β-subunit M2-M3 loops. However, the δ subunit M2-M3 loop is orientated inward and the 9’ activation gate in the pore is closed, consistent with the low efficacy for these receptors. Addition of a δ-selective positive allosteric modulator (PAM), DS2-Me, reveals binding to an α-δ pocket that causes the 9’ activation gate to open. In this work we provide key insights into the stoichiometry, arrangement and molecular modes of GABA activation and δ-selective modulation of these critical regulators of neuronal excitability.

## Abstract (web summary)

GABAA αβδ receptors regulate tonic inhibitory neurotransmission neuronal excitability and contribute to sleep, mood and motor coordination. Here, we show the subunit arrangement and molecular mechanism of modulation by a δ-subunit selective drug.

## Introduction

The neurotransmitter γ-aminobutyric acid (GABA) binds GABA_A_ receptors, which are pentameric ligand-gated ion channels (pLGICs), to open an intrinsic chloride channel. Structural, functional and pharmacological GABA_A_ receptor heterogeneity arises from different subunit combinations within the pentamer, with αβγ and αβδ receptors being the predominant synaptic and extrasynaptic isoforms respectively^1–4^. In the case of αβδ receptors, activation by GABA generates a membrane tonic conductance that sculpts neuronal excitability^4,5^. δ-containing isoforms contribute to sleep, mood and motor coordination, and dysfunction causes postpartum depression (PPD), epilepsy and ataxia^4,6–8^.

For αβγ receptors, two molecules of GABA bind, one at each β-α interface and tilt both β-subunit extracellular domains (ECDs) to outwardly displace the M2-M3 loops underneath and open a 9’ hydrophobic pore ring midway across the transmembrane domain (TMD)^9–13^. Beneath this a −2’ “desensitisation” gate at the intracellular end is closed in all structures determined so far^9,13–17^. For αβδ receptors, the mechanism of GABA transduction is less clear because extant structures assume an α-β-β-β-δ arrangement with a single GABA pocket and they are additionally bound by histamine at β-β pockets^15^. However, biophysical studies generally favour a 2α:2β:1δ stoichiometry and α-β-α-β-δ arrangement^18–22^, although variation in the arrangement may be possible^19,21,22^. Thus, αβδ receptors in an arrangement that possesses the classical two GABA pockets have not been structurally resolved despite evidence favouring this. Furthermore, whilst numerous γ-selective modulator structures have been determined^2,3,9,23–25^, δ-containing receptors have proven resistant to the development of selective PAMs, with DS2 (4-chloro-N-[2-(2-thienyl)imidazo[1,2-a]pyridin-3-yl]benzamide) being a rare example^26,27^. Currently, the molecular basis of δ-selective drug modulation is not understood and structural determination of αβδ receptors bound by δ-selective PAMs could enable structure-based drug design.

Here, we develop a technical pipeline utilising structural and pharmacological nanosensing fiducials to identify and resolve αβδ receptors with an α-β-α-β-δ arrangement, to observe a double GABA-bound conformation, and to uncover the molecular action of the δ-selective PAM, DS2-Me.

## Results

### αβδ stoichiometry follows an α-β-α-β-δ arrangement

Previous structures of α4β3δ receptors utilised Nb25, which recognises the β3-β3 interface, as a fiducial to align subunits within particles for map reconstruction^15,28^. To align α4β3δ particles lacking this interface requires alternative fiducials. From a panel of nanobodies previously raised against α1β3 receptors^28^ we identified an alternative β-specific nanobody, Nb24, which bound α1β3γ2 receptors lacking a β-β interface, in contrast to Nb25 which only bound β3 homomers (Supplementary Fig. 1a, b). To further confirm αβδ subunit arrangement (Supplementary Fig. 1c), we identified an additional fiducial against the α4 subunit. From the same panel we identified Nb30 which bound α1, but mutagenesis screening revealed that a single residue switch from α4 Met120 to the α1 Thr equivalent enabled binding to the α4-M120T subunit (Supplementary Fig. 1d, e). Whole cell patch-clamp recordings revealed that Nb24 does not modulate function, whilst Nb30 acts as an agonist on both α1β3γ2 receptors and the cryo-EM construct, α4^M120T^β3δ_EM_ (hereafter termed αβδ-EM) (Supplementary Fig. 1f–i). Two-electrode voltage-clamp recordings from oocytes expressing αβδ-EM, revealed robust GABA responses and an EC_50_ ~ 1 µM, comparable to previous reports^29^ (Supplementary Fig. 1j).

**Fig. 1:**
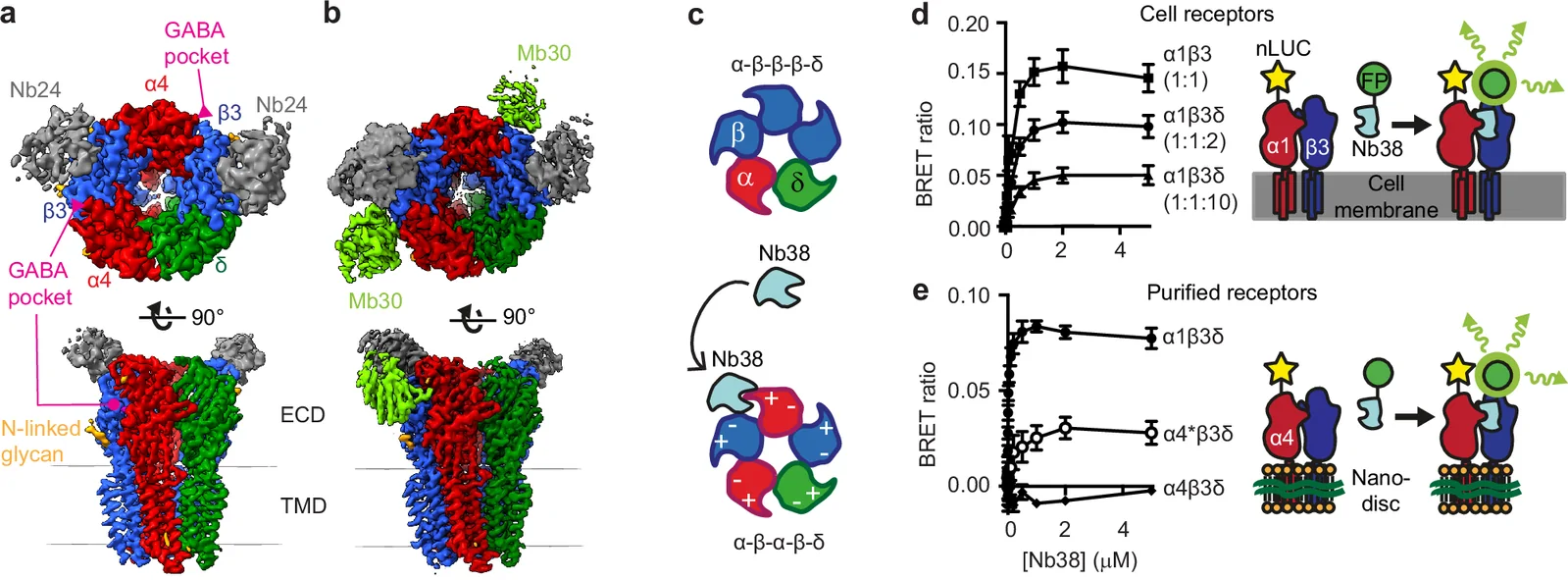
α4β3δ GABA_A_ receptor stoichiometry. **a**, **b** GABA-bound α4β3δ receptor cryo-EM maps, showing top (upper) and side (lower) views, with Nb24 fiducial or Nb24 and Mb30 fiducials, respectively. **c** Receptor schematic showing Nb38 binding an α(+)/β(-) interface that is only present in the α-β-α-β-δ arrangement. ‘+’ refers to the ‘principal’ face of the subunit containing loop-C; ‘-’ refers to the ‘complementary’ face of the subunit. **d** On-cell nanoBRET Nb38 binding curves for α1-containing receptors, demonstrating detection of the α-β interface. NanoBRET schematic of Nb38 with C-terminal monoVenus tag (fluorescent protein – FP) binding a nanoluciferase tagged GABA_A_ receptor. **e** Nanodisc purified protein nanoBRET binding curves (and schematic) demonstrating detection of the α-β interface in α1β3δ and α4*β3δ receptors, but not in the α4 wild-type negative control that cannot bind Nb38. Data are mean ± s.e.m. *n* = 4 for four independent experiments for each receptor type.

We determined the cryo-EM structure of αβδ-EM in an MSP2N2 nanodisc in the presence of 500 µM GABA, and bound by Nb24 at 3.3 Å resolution (Supplementary Fig. 2a, Supplementary Table 1). In contrast to the previous α-β-β-β-δ receptor structure^15^, Nb24 identifies only two β3 subunits in this receptor, which exhibits an α-β-α-β-δ clockwise stoichiometry (Fig. 1a). Data processing did not reveal any alternative arrangements even though this fiducial should theoretically identify them because it binds single β3 subunits (Fig. 1a). To further confirm this arrangement and discount impacts from batch variability a fresh sample was purified and the structure re-determined but this time also including the α4^M120T^ fiducial, Nb30, reformatted as a megabody, Mb30, to confirm α-subunit identity, and enhance particle tumbling to improve resolution^30^. Inclusion of both Nb24 and Mb30 also confirms δ subunit identity, because it is the only subunit lacking a fiducial marker. Resolution improved to 2.7 Å (Supplementary Fig. 2b and Supplementary Table 1). Mb30 is seen binding across two β3-α4 interfaces over the GABA binding pocket, confirming the same stoichiometry (Fig. 1b and Supplementary Fig. 2b). No alternative arrangements or conformations were detected during follow-up 3D classification. The primary variability observed between classes was limited to differences in local resolution and map quality within the TMD.

This structure matches the 2α:2β:1δ stoichiometry implied by previous biophysical reports^18–22^. To further examine this, we utilised a nanobody, Nb38^9^, specific for the α(+)/β(-) interface that is only present in α-β-α-β-δ receptors, and not in the α-β-β-β-δ arrangement^15^(Fig. 1c). Due to the low expression levels and purification yields of full-length αβδ receptors for cryo-EM and an inability to solve structures in cells to directly see Nb38 engagement, we instead performed nanoluciferase bioluminescence resonance energy transfer (nanoBRET) because of its high sensitivity to detect binding events at equilibrium both on cells and purified protein^31^. Nb38 is α1 selective^32^, therefore we tested full-length wild-type α1β3δ receptors which represent an alternative physiological isoform^33^. A clear nanoBRET signal was detected from cells expressing α1β3 receptors at 1:1 subunit transfection ratio, and for α1β3δ receptors even when the ratio of δ plasmid DNA in the transfection mixture was increased 10-fold over α1 and β3 to enforce the highest possible δ inclusion into pentamers (Fig. 1d). A nanoBRET signal was also clearly detectable using very low concentrations of purified α1β3δ receptors ( ~ 100 pM), which were purified against a purification tag only on the δ-subunit to ensure its inclusion in every pentamer (Fig. 1e). Furthermore, introduction to α4 of α1 binding residues to Nb38 created full-length α4*β3 receptors that bind Nb38 (Supplementary Fig. 3a). However, although a nanoBRET binding curve could be measured on cells expressing α4*β3 receptors, the maximum signal, nanoBRET_MAX_, was substantially reduced (nanoBRET ratio = 0.018 ± 0.003) versus α1β3 receptors (nanoBRET ratio = 0.16 ± 0.016)(Supplementary Fig. 3a). This was presumably due to the α4 subunit reducing surface expression, as verified by a streptavidin surface staining assay: α4β3 and α4*β3 fluorescence = 2700 ± 1200 AU and 1700 ± 950 AU respectively; versus α1β3 fluorescence = 6700 ± 950 AU, P < 0.001 (Supplementary Fig. 3b). In the strep staining assay full-length α4β3δ and α4*β3δ receptor signals were close to background (Supplementary Fig. 3b) and nanoBRET did not give reliable results (Supplementary Fig. 3c). To circumvent low cell expression of full-length α4β3δ receptors, we instead purified full-length α4*β3δ receptors so that we could control the concentration, and we used a purification tag only present on the δ-subunit to ensure its inclusion, and this approach did reveal clear binding by Nb38 (Fig. 1e). Overall, Nb38 confirms the presence of the β-α interface in full-length α1β3δ receptors whether expressed on cells or purified, and in full-length α4*β3δ receptors that have been purified, consistent with the observation of this interface in the structure of the cryo-EM construct.

### αβδ adopts a GABA-bound closed-channel conformation

**Fig. 2:**
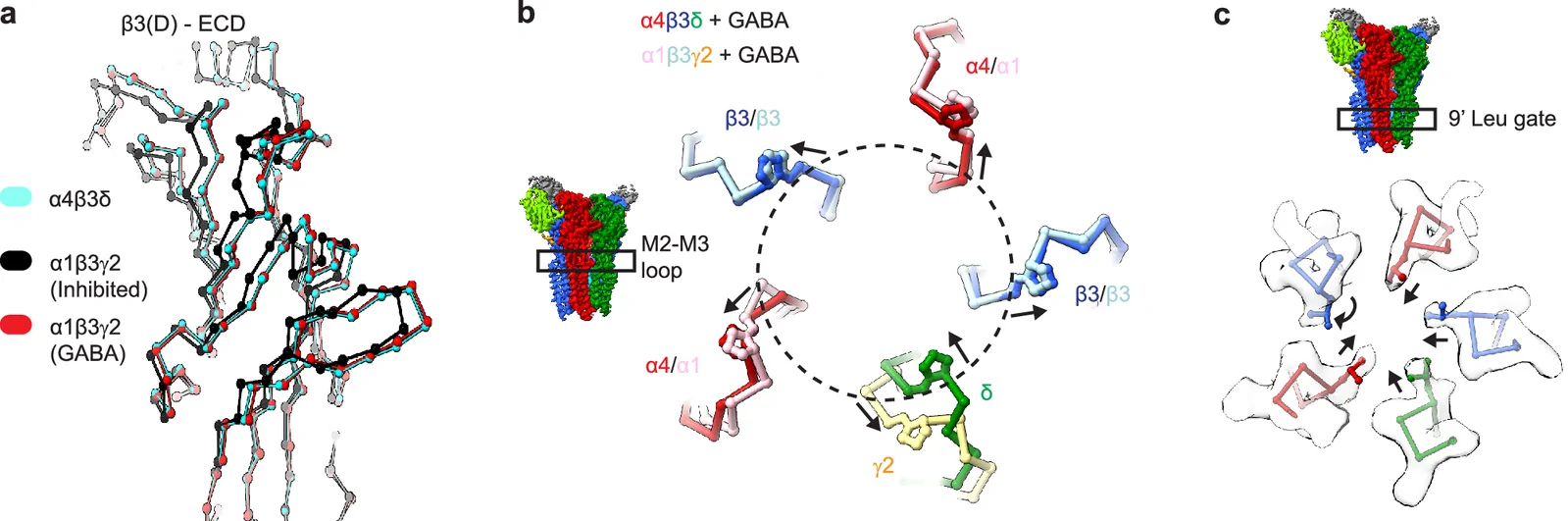
α4β3δ GABA_A_ receptor conformation in the presence of GABA. **a** Global structural alignment of α4β3δ-GABA versus α1β3γ2 receptors. The β-subunit ECD (chain D)(cyan) is tilted to assume the α1β3γ2-GABA (PDB:6HUO – red) conformation instead of the α1β3γ2-bicuculline (inhibited; 6HUK – black) conformation. **b** Top-down view of the M2-M3 loops showing a conserved proline as a positional marker, comparing α4β3δ-GABA with α1β3γ2-GABA. **c** Top-down cross-section of the α4β3δ-GABA pore model/map at the 9’ Leu ring with only one leucine rotated outwards (black curved arrow versus straight arrows).

Comparison of the structural models of αβδ-EM bound by GABA, Nb24 and Mb30 versus without Mb30 reveal identical conformations throughout (overall C_α_ RMSD 0.12 Å; 9′ Leu ring, −2′ ring, δ M2-M3 loop, β M2-M3 loops all <0.15 Å). Both models possess two GABA binding pockets at the β-α interfaces that contain GABA ligand non-protein density (Supplementary Fig. 4a-c). This contrasts with the previous α-β-β-β-δ receptor structure which contained a single GABA binding pocket and two histamine pockets^15^, and instead matches the set-up reported for α1β3 and α1β3γ2 receptors^9,14^. Given that the Mb30 model was built from a higher resolution map, this model is used for all further analysis. Both β-subunits have undergone the classical tilt expected in response to binding GABA, matching α1β3-GABA (PDB:7PBD; Cα RMSD 0.56 Å) and α1β3γ2-GABA (PDB:6HUO; Cα RMSD 0.48 Å), but not α1β3-CBTx inhibitor (PDB:7PCO; C_α_ RMSD = 1.55 Å), and α1β3γ2-bicuculline inhibitor (PDB:6HUK; C_α_ RMSD = 1.61 Å)(Fig. 2a and Supplementary Fig. 4d). Each β-subunit M2-M3 loop, which sits under the ECD at the top of the TMD helical bundle, is positioned outwards, matching the α1β3γ2-GABA conformation (Fig. 2b) rather than inward as in the αβγ-inhibitor structure (Supplementary Fig. 4e,f). Notably however, the δ-subunit M2-M3 loop remains inward, distinct from the outward position of the γ2-subunit (Fig. 2b). This δ-subunit configuration matches the α-β-β-β-δ receptor bound by one GABA molecule and two histamine molecules (Supplementary Fig. 4g), rather than the one bound by three histamine molecules for which the δ-subunit M2-M3 loop is even further inward (Supplementary Fig. 4h). The 9’ activation gate that exists midway down the pore as a ring of five M2 helix leucine residues reveals that despite the activated β3 ECDs and M2-M3 loops, only the β3 leucine non-adjacent to δ has rotated out of the pore, resulting in an asymmetric gate (Fig. 2c). This limits the gate diameter to 3.3 Å (Supplementary Fig. 4i), which is not wide enough to conduct chloride anions (Pauling radius 1.8 Å). This contrasts with the open 9’ gate in the GABA-bound α1β3γ2 receptor (5.4 Å) with all leucines rotated outwards^9^ and is much narrower than in the αβββδ receptor bound by one GABA and two histamines (7.1 Å)^15^. We hypothesise that the inward position of the δ-subunit M2-M3 loop limits the transduction effect through the neighbouring β3 TMD. Observation of a closed 9’ activation gate is consistent with our single channel recordings from αβδ receptors showing that saturating GABA induces mainly individual openings and only few infrequent brief bursts, giving a low open probability (P_o_) of 0.14, and within bursts P_o_ = 0.64 (Supplementary Fig. 4j–r). This means openings will not be observed in the ensemble averaging of the Cryo-EM structures. Regarding the −2’ “desensitisation” gate at the intracellular end of the pore, this assumes the typical closed conformation observed in all structures reported so far (Supplementary Fig. 4i)^9,14–16,25^.

### Molecular mechanism of δ-selective positive allosteric modulation

**Fig. 3:**
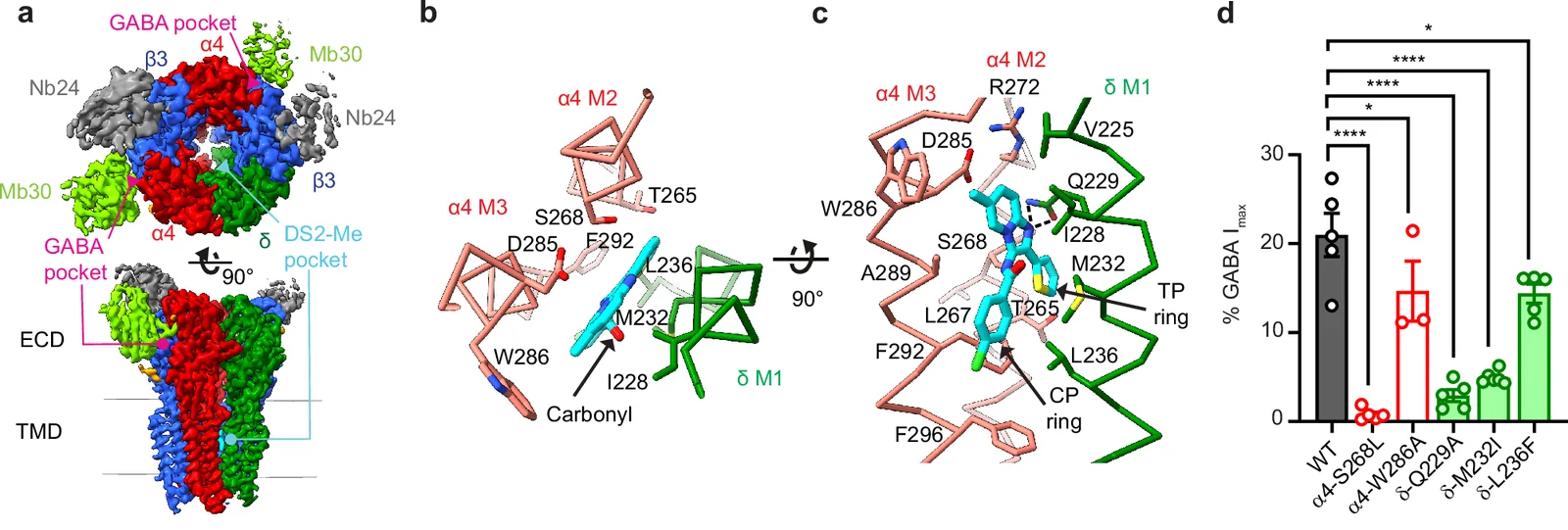
DS2-Me bound at the α4/δ interface. **a** Cryo-EM map of the αβδ-GABA-DS2-Me complex, showing top (upper) and side (lower) views. **b** Top-down view of the DS2-Me binding pocket and interacting residues (cutoff <4 Å). **c** Equivalent side-on view. DS2-Me – cyan, oxygen – red, nitrogen – blue, sulphur – yellow, chlorine – lime, TP – thiophene, CP – Chlorophenyl, putative polar interactions – dashed lines. **d** Oocyte recording potentiation of EC_1-3_ GABA responses by 1 µM DS2-Me on α4β3δ receptors that are wild-type (WT) or contain α4 or δ mutations (red open bars represent α4-subunit mutations; green lightly shaded bars represent δ-subunit mutations). Data are mean ± s.e.m. (WT *n* = 5, α4-S268L *n* = 5, α4-W286A *n* = 3, δ-Q229A *n* = 5, δ-M232I *n* = 6, δ-L236F *n* = 5). Each *n* = 1 is a recording from a different oocyte. One-way ANOVA Dunnett’s multiple comparisons; * = *P* < 0.05; **** = *P* < 0.0001. WT versus: α4-S268L, *P* < 0.0001; α4-W286A, *P* = 0.0438; δ-Q229A, *P* < 0.0001; δ-M232I, *P* < 0.0001; δ-L236F, *P* = 0.0123.

We determined the structure of α4β3δ-GABA-Nb24-Mb30 in the presence of the δ-selective PAM, DS2-Me^27^ to 3.0 Å resolution (Supplementary Fig. 2c, Supplementary Table 1). Previous reports have proposed a binding mode for DS2 between the α-δ ECD interface or between the α-β TMD interface^27,34^. However, the structure instead shows non-protein density that accommodates DS2-Me only at the α-δ TMD interface (Fig. 3a and Supplementary Fig. 5a-c), which is absent from the GABA alone map. Notably, this contrasts with αβγ TMD PAM structures that show a common theme of two pockets for the bound molecules, such as general anaesthetics etomidate and propofol, the barbiturate phenobarbital, and endogenous neurosteroids^25,35^. DS2-Me is chemically unrelated to these molecules and forms a planar aromatic slice between the α4 M2/M3 helices and the δ M1 helix with the chlorophenyl ring pointing towards the external lipids (Fig. 3b, c and Supplementary Fig. 5a–d). Consistent with the ligand density curvature, the adjoining carbonyl also points out from the pocket (Fig. 3b, c and Supplementary Fig. 5c). An indentation in the ligand electron density identifies the thiophene ring to be positioned deepest in the pocket facing the back of the pore-lining M2 helix (Fig. 3b, c and Supplementary Fig. 5a). DS2-Me forms extensive vdW contacts across the α-δ interface, with α4 M2 S268, α4 M3 A281, D285, W286, A289, and δ M1 I228, Q229, M232, P233 and L236, all within 3 Å (Fig. 3b, c). Substitution of α4 M2 S268 with a larger leucine side chain so that it will sterically clash with DS2-Me^34^ (Fig. 3b) completely ablated potentiation of GABA EC_1-3_ responses (Fig. 3d, Supplementary Fig. 5e,f). In addition, a δ Q229A substitution to remove putative polar and vdW interactions with the imidazole ring, reduced potentiation by ~75 % (Fig. 3d, and Supplementary Fig. 5e). Two peripheral pocket mutations, α4 W286A and δ L236F, exerted more modest but statistically significant reductions in potentiation (Fig. 3d, Supplementary Fig. 5e). Notably, the cryo-EM construct exhibited comparable DS2-Me sensitivity and levels of potentiation to wild-type receptors (Supplementary Fig. 5g). The αβγ receptor possesses the same residue identities in the DS2-Me pocket to αβδ receptors except for an Ile at the equivalent position to δ M232 (Supplementary Fig. 5h). An M232I substitution in δ reduced potentiation ~5-fold suggesting a branched aliphatic moiety at this position less favourably accommodates ligand binding, presumably due to its added bulk (Fig. 3d; Supplementary Fig. 5e). As well as the M232 residue difference, the upper γ2 M1 helix is rotated further than for δ, which changes the position of the Ile-Gln residue pair, causing the γ2 Ile to sterically clash with DS2-Me, and the γ2 Gln to lose favourable contacts (Supplementary Fig. 5i). Together, these differences likely explain the mode of DS2-Me δ-selectivity.

**Fig. 4:**
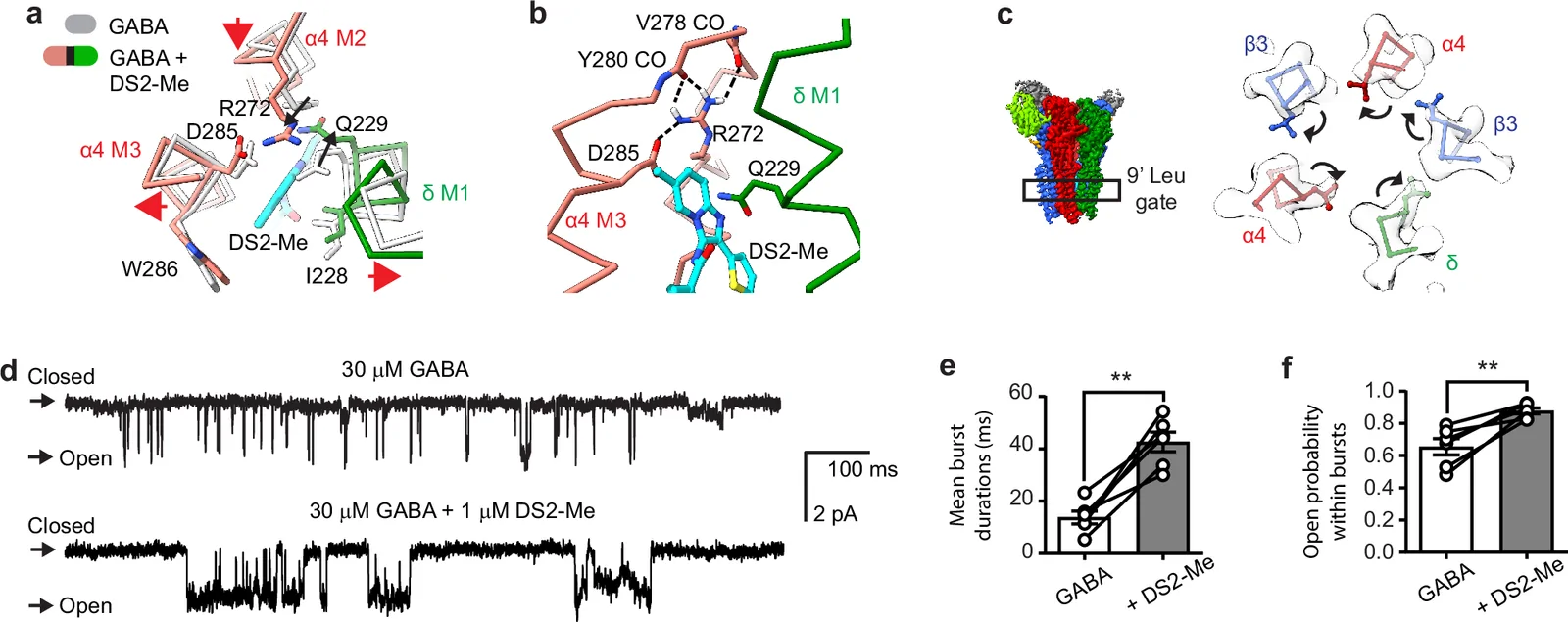
DS2-Me mechanism of action. **a** Global structural alignment of α4β3δ bound by GABA (grey) versus GABA + DS2-Me (salmon/green). DS2-Me – cyan, oxygen – red, nitrogen – blue, pocket expansion (red arrowheads), Q229/R272 rotamer switches (black arrows). **b** Repositioned R272 putative polar/salt-bridge interactions (dashed lines, <3 Å). **c** 9’ ring cross-section of α4β3δ-GABA-DS2-Me model/map with leucines rotated outwards. **d** Example outside-out patch single channel recordings of α4β3δ receptor induced by 30 μM GABA without versus with 1 μM DS2-Me. Burst durations, **e** and open probabilities within bursts, **f** significantly increase in the presence of DS2-Me (paired two-tailed *t* tests; ** = *P* < 0.01, respectively). Burst duration GABA versus GABA + DS2-Me, *P* = 0.001; open probabilities within bursts GABA versus GABA + DS2-Me, *P* = 0.005). Data are mean ± s.e.m., *n* = 6 independent single channel recordings from separate cells.

Structural alignment in the absence versus presence of DS2-Me reveals that the pocket expands between α4 M3 and δ M1 to accommodate binding (Fig. 4a). To avoid ligand clashes, the δ Q229 rotamer is repositioned deeper in the pocket, displacing the α4 M2 R272 side chain upwards (Fig. 4a). This results in R272 creating an additional network of three H-bonds and one salt-bridge, all <3 Å, between M2, the M2-M3 loop at the V278 and Y280 peptide carbonyls, and M3 at the D285 side chain carboxyl (Fig. 4b). This pulls the M2 helix towards the DS2-Me ligand and away from the pore (Fig. 4a). Substitution of R272 with alanine to remove charge and bulk or glutamine to remove charge but retain some polarity and bulk, both resulted in substantial, >90 %, reductions in GABA I_max_ (100 µM) from 148 ± 43 nA to 8 ± 2 nA and 13 ± 2 nA respectively (*n* = 3), presumably due to an impact on gating, assembly or protein folding. These small responses from receptors containing the R272 mutations precluded measurement of DS2-Me modulation of GABA EC_1-3_ responses and prevent direct confirmation of any role it might have in the DS2-Me PAM effect due to its apparent importance in general protein function. The local effect on the R272 network does not alter the M2-M3 loop positions versus the GABA alone structure (Supplementary Fig. 6a, b), but the α4M3-δM1 expansion and M2 pull from the pore substantially impacts the 9’ activation gate, resulting in all five 9’ Leu side chains being rotated outwards, increasing the gate diameter from 3.3 Å for the GABA alone structure to 10.8 Å (Figs. 2c, 4c and Supplementary Fig. 6c, d), sufficiently wide for chloride flux. Notably, in contrast to structures of αβγ receptors bound by TMD PAMs^25,35^, which were solved in the presence of GABA which by itself is sufficient to open the 9’ gate, for the αβδ receptor it is the TMD PAM itself that is required to induce gate opening because GABA alone is less effective (Supplementary Fig. 7a, b). To have captured this transition structurally suggests that DS2-Me has increased the likelihood (probability) of the 9’ gate assuming the open conformation. Single channel recordings from wild-type α4β3δ receptors confirmed that DS2-Me increases mean burst duration >3-fold, and the burst P_O_ increased from ~0.64 to ~0.89 (Fig. 4d–f and Supplementary Fig. 6e). The duration of brief openings (τ1) was unchanged but the duration of longer openings (τ2) increased as did the proportion of entries into longer open states (A2) versus brief open states (A1), increasing the P_O_ ~ 2-fold (Supplementary Fig. 6f–m). In contrast, channel current amplitude, and so conductance, was unaffected (Supplementary Fig. 6n). Again, as previously mentioned for the GABA alone conformation, the -2’ desensitisation gate was closed, consistent with prolonged agonist exposure and comparable to previous αβγ structures (Supplementary Fig. 6d).

## Discussion

We solve α4β3δ receptor structures revealing an α-β-α-β-δ arrangement. Separately, we also use nanoBRET nanosensors to support the presence of an α(+)/β(-) interface in full-length versions of both α1β3δ and α4β3δ receptor isoforms, which are absent from the previously determined α-β-β-β-δ subunit arrangements but will exist in our α-β-α-β-δ subunit arrangement. These receptors contain two GABA binding pockets and adopt a GABA-bound closed 9’ gate associated with low efficacy measured by electrophysiology. Previously, receptors with an alternative α-β-β-β-δ arrangement were prepared from stable cell lines and a β-β selective Nb fiducial was used^15^, which can only identify pentamers containing this interface. In that study, two alternative ratios of α4 to β3 subunit were used, both favouring α4 plasmid DNA, being α4:β3:δ = 2:1:0.25 or 2:0.3:0.25. This suggests it should have been possible to induce the formation of pentamers containing 2 copies of the α-subunit, that match our structures. However, stable cell lines rely on relatively low copy number inclusion into the genome making relative expression a stochastic process. For example, at those ratios many cells might incorporate one copy of the α4-subunit DNA and one copy of either the β-subunit or the δ-subunit DNA but not both. This would result in either α4β3 receptors being formed in cells which would fail to purify due to lack of a purification tag, or α4-δ subunit combinations forming in cells which are not known to form functional receptors and which might also fail to be purified. Whilst in instances where cells did incorporate one or more copy of all three DNAs into the genome, enabling the formation of α4β3δ receptors that can be purified, expression would be unlikely to equate to the input DNA transfection ratio due to stochastic genome insertion and further complications from different insertion locations yielding unequal expression. Finally, the relative quantity of δ-subunit DNA is very low in the previous study, being less than 10 % of the total DNA, compared to the amount used in electrophysiology studies typically being above 50 % to ensure its incorporation into all pentamers. Given the apparently broad assembly landscape of α4β3δ receptors, in situations where the δ-subunit is scarce this might favour alternate stochiometric arrangements versus situations where the δ-subunit is more abundant, as is the case in our study which included the δ-subunit at 30 % of total DNA. Our transient expression system enables control of plasmid transfection quantities in a non-stochastic manner due to the absorption of hundreds to thousands of copies of plasmids per cell. These are all under equal expression drive, being expressed from separate identical pHLsec plasmids with the same promoters, kozak sequences and signal peptides. This better supports inclusion of the α-subunit at a controlled ratio, and we combined this with Nb fiducials that can detect pentamers lacking the β-β interface to ensure such pentamers are not excluded from processing at the 2D class stage due to the lack of fiducial earmarks. In our study, were receptors with β-β interfaces present, we should have detected them, however, as was the case with the alternative study, we also used a higher DNA ratio of the α4 subunit, being α4:β3:δ = 1.25:1:1, which might have been sufficient to preclude pentamers containing neighbouring β3-subunits. Of note, this arrangement is consistent with previous biophysical studies favouring a 2α:2β:1δ stoichiometry and α-β-α-β-δ arrangement^18–22^. Furthermore, our arrangement provides a plausible molecular explanation for the reduced efficacy that is observed for α4β3δ receptors by electrophysiology versus α4β3γ2 receptors, due to the δ-subunit M2-M3 loop adopting an inward conformation versus the outward conformation of the γ2-subunit M2-M3 loop, which coincides with the 9’ gate being closed even with GABA bound. This contrasts with the previous α4β3δ receptor structures in which the 9’ gate is open regardless of the type or number of agonist molecules bound^15^. Overall, whilst both studies demonstrate plausible αβδ arrangements, future studies should aim to reveal the physiological arrangement(s).

We also characterise a DS2-Me TMD PAM binding pocket between the α4 and δ-subunits. DS2-Me binding at this single pocket is sufficient to convert the GABA-bound closed 9’ gate conformation to an open gate conformation. This is distinct from the effects of the αβγ PAMs that bind in the TMD, such as the intravenous anaesthetics, propofol and etomidate, and the barbiturates and neurosteroids, which all bind two separate pockets^25,28,35,36^ (Supplementary Fig. 7a, b). Two previous studies on the analogue DS2, which differs from DS2-Me only by the loss of a single methyl group, have each proposed their own alternative binding sites: one being between the α4 and δ-subunit ECDs at a homologous pocket to the neurotransmitter site^27^; the other binding in the TMD at the α4–β1 interface^34^. In the case of the proposed ECD pocket this was based on computational docking using a homology model from the α1β3γ2 subtype (PDB:6D6T), which differs from experimental δ-containing structures^27^. Additionally, the authors acknowledged that their putative binding site proposal could not explain the structure-activity relationship (SAR) around the thiophene substituent on the imidazo-pyridine core of DS2 or DS2-Me. Arguably, our structure provides evidence for the SAR on position 2 of the imidazo-pyridine core. This is based on the size of a small lipophilic pocket where the thiophene resides, alongside structural requirements for ligand geometry (specifically, the full planarity of the imidazo-pyridine core and the thiophene ring) that are not fulfilled when larger rings, such as a phenyl group, replace the thiophene. In the case of the proposed α4-β1 TMD pocket, the data was based on two mutations, α4(S303L) and β1(I289Q), which markedly reduced DS2 modulation^34^. We show here that S303 is also present in our α4-δ TMD pocket (mature numbering S268), and we also observed that a leucine mutation ablates DS2-Me modulation (Fig. 3d). Given that S268 is important for both DS2 and DS2-Me modulation (shown in previous study and this study respectively) we propose both ligands bind the same α4-δ pocket determined here using cryo-EM, and that the β-subunit mutation might exert an indirect effect on modulation. Of note, our identified pocket fully explains the SAR around the 5-position of imidazo-pyridine ligands, as well as the increased potency of DS2-Me compared to DS2^27^. This is based on an additional interaction of the methyl residue at the 5-position with α4-W286. This finding is further supported by the drop in potency recorded for DS2-Me in the α4-W286A mutant (Fig. 3d). Overall, this DS2-Me pocket is capable of engendering δ-selective binding/modulation and therefore represents a target for drugs to treat postpartum depression, ataxia and epilepsy disorders in the future.

## Methods

### Ethical statement

Ovaries were harvested from Xenopus laevis female frogs following procedures established with the Geneva Canton agreement with the authorization from the animal care 34485 GE177. Frogs were obtained from the TEFOR PARIS SARCLAY Institut des Neurosciences, bât. 151, 151 route de la Rotonde 91400 Sarclay, France.

### Compounds used in the solved protein structures in this study

GABA was purchased from Merck (Sigma-Aldrich). DS2-Me was synthesised and provided by Roche.

### GABA_A_ receptor construct design

For electrophysiology human GABA_A_ receptor α1/α2/α4/β3/γ2/δ subunit wild-type mature protein sequences were used (PDB IDs - α1 Uniprot P14867 entry Gln28 is Gln1 1-429 QPSL…PTPHQ; α2 Uniprot P47869 entry Asn29 is Asn1 1-423 NIQE…LGVSP; α4 Uniprot P48169 entry Gln36 is Gln1 1-519 QNQK…SESLM; β3 Uniprot P28472 isoform 1 entry Gln26 is Gln1 1-448 QSVN…LYYVN; γ2 Uniprot P18507 isoform 1 entry Gln40 is Gln1 1-428 QKSDD…SYLYL; δ Uniprot O14764 entry Met25 is Met1 1-428 MNDI…AAYAM). For nanoBRET experiments, constructs were the same as electrophysiology experiments except for the following differences: an N-terminal nanoLuciferase (nLUC; VFTLE…ERILA)^37^ (FPbase ID: M8P3S) was added on each subunit for nanoBRET experiments prior to the Age restriction site; an N-terminal streptavidin binding protein (SBP) purification tag^38^ (MDEKTTGWRGGHVVEGLAGELEQLRARLEHHPQGQREP), followed by a flexible GGSGGS linker before the nLUC, on the δ construct; for α4* the following amino acid substitutions were introduced into the α4 construct to match α1: H215L, D216A, G287A, L288H, G289A, Q290A, T291D, L292S, D293G, I294V. To boost expression and purification yields for cryo-EM, constructs were modified to substitute the intracellular M3-M4 trafficking loop with either a glvi linker sequence SQPARAA or a modified glvi sequence containing the *Escherichia coli* soluble cytochrome B562RIL41 (BRIL, amino acids 23–130, ADLE…QKYL, Uniprot P0ABE7) to give the sequence SQPAGT-BRIL-TGRAA^14^. Linker regions substituted were: α4 between Ile311-Ser481; β3 Gly308-Asn421; δ Asp312-Asp401. All constructs were subcloned into pHLsec^39^ directly after the pHLsec signal peptide between Age/Kpn or Age/Xho restriction sites.

### Nanobody and megabody construct design

Nanobodies (24;30;38) were subcloned into the aforementioned pHLsec vector between the N-terminal signal sequence and a C-terminal His6 purification tag, or between the N-terminal signal sequence and a C-terminal monoVenus-OctaHis (MVSKG…DELYK) (FPbase ID: WCSN6)^40^ tag depending on experimental application, corresponding to insertion between Age/Kpn or Age/Xho restriction sites. The VHH domain begins with the conserved N-terminal motif QVQLQESG and ending in C-terminal QVTVSS for each nanobody. The megabody construct Mb30 was cloned into the pBAD expression vector under control of the arabinose-inducible promoter, with an N-terminal *pelB* leader sequence for periplasmic targeting and C-terminal His₆–EPEA tags for purification and detection. The Mb30 scaffold was based on a previously described megabody framework^30^.

### Nanobody and megabody expression and purification

For nanobody production: Expi293F (Thermofisher, A14527) cells were cultured in either BalanCD HEK293 medium (*Fujifilm Irvine Scientific*), supplemented with 5 mM L-glutamine, or Expi293 Expression Medium. Cells were transfected at 4 × 10^6^ cells/ml using 2 mg plasmid DNA and 8 mg PEI Max per 1 mL of culture. 18 hrs post-transfection cells were centrifuged at 300 g for 5 minutes, media was discarded, and pellets were resuspended in double the volume of fresh media. After 5-7 days of expression, cells were pelleted and the supernatant stored at 4 °C until needed. Nanobodies possessed C-terminal HexaHis or OctaHis tags and were purified using Ni-NTA agarose beads (Qiagen, 30210). Per 12 ml of harvested media, 1 ml binding buffer containing 500 mM Tris pH 7.8, 3 M NaCl was mixed with 0.2 mL bead slurry and added to the media. After gentle rotation at 4 °C for 1 h, the beads were recovered and washed twice, first with ~50 CVs of phosphate buffered saline (PBS) containing 10 mM Tris-HCl pH 8.0, 0.05% (v/v) Tween-20, and then with ~50 CVs PBS containing 10 mM Tris-HCl pH 8.0, 5 mM imidazole. Elution was performed using 2–3 CVs of buffer containing 500 mM NaCl, 20 mM Tris-HCl pH 8.0, 250 mM imidazole. Imidazole was removed by concentration dialysis or size exclusion chromatography, and protein concentrated (Amicon, 10 MWCO) to ~100–500 µM prior to snap-freezing and storage at −80 °C.

Mb30 was expressed in *E. coli* grown in Terrific Broth supplemented with 0.02% L-arabinose and harvested by centrifugation. Cell pellets were resuspended in lysis buffer (20 mM Tris-HCl pH 7.0, 150 mM NaCl, 10 mM imidazole) supplemented with EDTA-free protease inhibitor (Roche), 1 µL/50 mL benzonase (Sigma), 1 mM MgCl₂, and 1 mg/mL lysozyme. Cells were homogenised and disrupted using a high-pressure microfluidizer at 1200 bar. Lysates were clarified by centrifugation at 70,000 × *g* for 1 h at 4 °C. The supernatant was loaded onto a 1 mL HisTrap HP column (Cytiva) pre-equilibrated in lysis buffer using an ÄKTA system. The column was washed with wash buffer (20 mM Tris-HCl pH 7.0, 150 mM NaCl, 20 mM imidazole) until baseline UV absorbance was reached. Bound protein was eluted with elution buffer (20 mM Tris-HCl pH 7.0, 150 mM NaCl, 300 mM imidazole) using a linear gradient over 20 min. Elution fractions were analysed by SDS-PAGE (NuPAGE 4–12% Bis-Tris gel; InstantBlue staining) and pooled. The pooled eluate was dialysed (20 kDa MWCO) into SEC buffer (20 mM Tris-HCl pH 7.0, 150 mM NaCl), concentrated to ~1 mL using centrifugal concentrators (Amicon Ultra, 3 kDa MWCO), and subjected to size-exclusion chromatography (SEC) on a Superdex column equilibrated in SEC buffer. Peak fractions were analysed by SDS-PAGE, pooled, and concentrated to ~4 mg/mL. Aliquots were flash-frozen in liquid nitrogen and stored at −80 °C.

### α4β3δ protein expression for Cryo-EM

Expi293F-GnTI- cells (Thermofisher, A39240), which yield proteins with truncated N-linked glycans (Man_5_GlcNAc_2_), were grown in suspension to densities of 4 × 10^6^ cells/ml in Expi293 Expression Medium (Invitrogen), shaking at 130 rpm, 37 °C, 8% CO_2_. Per 1 litre of culture, 3 mg polyethyleneimine (PEI) Max (Polysciences) and 1 mg plasmid DNA were added. Plasmids encoding α4-M120Tglvi, β3bril and SBP-δglvi, were co-transfected at a 1.25:1:1 ratio. Seventy-two h post-transfection, cell pellets were harvested, snap-frozen, and stored at -80 °C.

### GABA_A_ receptor expression screening

Expi293F-GnTI^-^ cells were cultured as above on a 30 mL scale and cell pellets following the same detergent solubilisation steps described below. Solubilised material was incubated with either streptavidin Agarose (for SBP-tagged constructs) or CNBr-activated sepharose beads (GE Healthcare) pre-coated with Rho-1D4 antibody (British Columbia)^41,42^ (for 1D4-tagged constructs) for 1 h at 4 °C with gentle rotation. Resin was washed with buffer B (as below) and bound protein was eluted with 150 mM NaCl, 10 mM HEPES pH 7.6 containing 0.01% LMNG10:1CHS  (buffer C) supplemented with either 10 mM biotin (SBP) or 1.5 mM 1D4 peptide overnight at 4 °C. Eluates were analysed by size exclusion- chromatography on a Superose 6 Increase 10/300 GL column equilibrated in buffer C to assess monodispersity.

### α4β3δ receptor purification and nanodisc reconstitution for Cryo-EM

Before purification, cell pellets were prepared as membrane pellets by resuspending on ice in buffer containing 20 mM HEPES pH 7.2, 300 mM NaCl (buffer A) supplemented with 1 % (v/v) mammalian protease inhibitor cocktail and sonicating (Fisherbrand Model 505 Sonicator; 13 mm probe, 500 W, 9 min, 6 s on/off pulses, 40% power). Insoluble material was removed by centrifugation (8000 g, 10 min), and the supernatant was ultracentrifuged (130,000 g, 2 h). The resultant membrane pellets were homogenised in buffer containing 20 mM HEPES pH 7.2, 300 mM NaCl, snap-frozen in liquid nitrogen, and stored at −80 °C. The membrane pellets were resuspended on ice in buffer A supplemented with 1 % (v/v) mammalian protease inhibitor cocktail. Membrane pellets were solubilised using 1% (w/v) lauryl maltose neopentyl glycol (LMNG; Anatrace)10:1 cholesterol (CHS, Anatrace) for 1 h at 4 °C, then centrifuged for 30 min at 10,000 *g* (4 °C) to remove insoluble material, following addition of buffer A to reduce detergent concentration to 0.6% (w/v). The supernatant was incubated with Pierce High Capacity Streptavidin Agarose (ThermoFisher, 20357) for 2 h at 4 °C. The resin was recovered via centrifugation at 300 g for 6 min and washed with 50 mL buffer B (buffer A containing 0.1% LMNG10:1CHS), followed by an incubation with buffer B supplemented with 10 mM ATP/MgCl_2_ for 30 min at 4 °C. The resin was recovered via centrifugation at 300 g for 6 min and finally washed with buffer B to remove excess 10 mM ATP/MgCl_2_. For on-bead nanodisc reconstitution, the streptavidin resin was equilibrated for 30 minutes in 1 ml DL buffer (detergent-lipid buffer with 20 mM HEPES pH 7.2, 300 mM NaCl, 0.1% (w/v) LMNG10:1CHS, containing phosphatidylcholine (POPC, Avanti) and bovine brain lipid (BBL) extract (type I, Folch fraction I, Sigma-Aldrich) mixture (POPC:BBL ratio 85:15 and final concentrations of ~3.5 mg/ml and ~1.3 mg/ml, respectively). Excess DL buffer was removed and 100 µL of MSP2N2 at 5 mg/ml together with Bio-Beads (40 mg/ml final concentration) and incubated for 2 h rotating at 4 °C. The MSP2N2 belt protein was produced as previously described^43^. After nanodisc reconstitution, the streptavidin resin and Bio-Bead mixture was washed extensively with buffer (300 mM NaCl, 50 mM HEPES pH 7.6) to remove empty nanodiscs. Receptors were eluted with 75–100 µl 75 mM NaCl, 12.5 mM HEPES pH 7.6, 10 mM biotin, overnight with gentle rotation at 4 °C. The next day, beads were centrifuged, and the eluate was collected and used directly for cryo-EM grid preparation.

### Cryo-EM sample preparation

Before freezing, all samples were deglycosylated with 0.01 mg ml^−1^ endoglycosidase F1 for 1 h at room temperature. Respective samples were incubated with nanobody and/or megabody to facilitate particle alignment and improve orientation distribution for 30 mins prior to freezing. Fiducials included 5 μM Nb24, 5 μM Nb30, 2.6 µM Mb30, 3 µM MbF3^44^. Ligands were also added at this time to respective samples including, 500 µM GABA and 100 M DS2-Me. Following incubation, 3 μl volume of sample was applied to double glow discharged UltrAuFoil R1.2/1.3 on 300 mesh Gold grids (Agar Scientific) and plunge frozen in liquid ethane using a Vitrobot Mark IV (FEI Thermo Scientific) operated at 4 °C, blot time of 3 seconds and 95% humidity. Grids were subsequently stored in liquid nitrogen.

### Cryo-EM data collection

Cryo-EM datasets were collected on Titan Krios G3 microscopes at the Department of Biochemistry EM facility (BiocEM, University of Cambridge) or eBIC, Diamond light source in electron counting mode at 300 kV. Microscopes are equipped with TFS Falcon 4i detector and Selectris X energy filter or Gatan K3 cameras and Gatan BioQuantum energy filters respectively. All datasets were acquired automatically using EPU software (Thermo Fisher Scientific, version 3.7–3.9). Details of data collection are summarised in Supplementary Table 1.

### Cryo-EM image processing

A general image processing workflow is shown in Supplementary Fig. 2. Motion correction, contrast transfer function correction, and particle picking were performed using either Warp1.0.9^45^ for collections obtained from Department of Biochemistry Cryo-EM facility, or cryoSPARC (from version 4.4.1 to 4.7.1) for data collections obtained at eBIC^46^. All subsequent particle processing was done in cryoSPARC. Extracted particles were subjected to 2D classification and good classes selected as inputs to generate three ab-initio models. The ab-initio 3D model with the most particles was subjected to homogenous refinement and non-uniform refinement to generate a refined model as reference. Subsequently all extracted particles were included in three or more iterative rounds of heterogenous refinement using five junk classes, generated from ab-initio reconstruction of unselected particles from earlier 2D classification rounds, and the refined receptor model. Each round of heterogenous refinement used only the particles associated with the refined model as input. The resulting 3D model and associated particles was further refined using homogeneous refinement and finally non-uniform refinement. For the final reconstructions, the overall resolutions were calculated by Fourier shell correlation (FSC) at 0.143 cutoff. Local resolution maps were also generated in cryoSPARC based on the FSC output calculated for voxels only within the mask output from the non-uniform refinement job used as the input for local resolution estimation. To generate maps coloured by local resolution, the local resolution map along with the main map was opened in UCSF ChimeraX (version 1.8)^47^ and processed using the surface colour tool.

### Model building, refinement, validation, analysis and presentation

Atomic models were built in COOT (version 0.9.8.8)^48^ using previously determined structures as references (PDB:7QN7). The individual chains were then modified to match our construct design. Small-molecule ligands and their geometry constraint files were generated using the Grade Web Server (Global Phasing)^49^. Initial Nb24 and Mb30 models were generated using AlphaFold2^50^ and then refined within respective maps using Coot. Map resolutions were sufficient to allow ab-initio building of the SQPARAA M3-M4 linkers. The larger and more disordered SQPAGT-BRIL-TGRAA M3-M4 linkers for the β3 chains were not modelled. Model refinement was performed through iterative rounds of local/manual inspection and global real-space refinement, using Coot and Phenix (version 1.21.1)^51^, respectively. Model geometry was evaluated using the MolProbity Web Server^52^ and the built-in Phenix validation tool. Measurements of pore diameters were generated using the HOLE plug-in in Coot^53^. Structural overlays were generated using Matchmaker function in UCSF chimera (version 1.8)^47^ and C_α_ RMSDs measured using the rmsd function. Structural presentations for figures were produced using UCSF Chimera. Model building and refinement statistics are shown in Supplementary Table 1.

### On-cell GABA_A_ receptor nanoBRET assay

Expi293F (Gibco) cells were cultured in either BalanCD HEK293 medium (*Fujifilm Irvine Scientific*), supplemented with 5 mM L-glutamine, or Expi293 Expression Medium. Cells were transfected with GABA_A_ receptor isoforms as described previously, volumes modified for 3 mL scale at 37 °C, 8% CO_2_, 70% humidity, shaking 250 rpm for 24 hr. Transfected cells were harvested via centrifugation at 1100 g for 3 min. The supernatant was discarded, and cells were gently resuspended in 5 mL PBS. Cells were centrifuged for a further 3 minutes at 1100 g and resuspended in 1 mL PBS. Nanobody titrations were prepared ranging from 0–5000 nM, diluted in PBS/0.5% BSA supplemented with 200 µM GABA. 50 µL  of each concentration was pipetted into the corresponding well of a LUMITRAC/FLUOTRAC white 96-well plates (Greiner, 655074). Approximately ~ 42,500 cells/well in 50 µL was added to each well alongside coelenterazine-h (NanoLight Technologies, 301) at a final concentration of 5 µM. Wells were pre-soaked with PBS/0.5% BSA prior to the experiment. A Mithras LB 940 Plate Reader (Berthold Technologies) was then used to obtain dual bioluminescence and fluorescence readings per well, measured at 460 nm and 530 nm, respectively. Readings were taken over a time period of 33 minutes, at 120 s intervals. Binding curves were fit using a one-site binding equation Y=B_max_ [Nb38]/(K_d_ +[Nb38]) in GraphPad Prism (version 10.4). Background-subtracted nanoBRET ratios were used for all analyses.

### Purified GABA_A_ receptor nanoBRET assay

Full-length GABA_A_ receptor isoforms were expressed on a 200 mL scale under the same conditions as described above and purified using the same nanodisc reconstitution method described above. Elutions were snap-frozen in 5 µl aliquots and stored at −80 °C. Purified receptors were then benchmarked to determine the receptor concentration required to yield raw bioluminescence signals in the range 20,000–100,000 RFU. Serial dilutions of each receptor isoform identified 0.3–0.5 nM as an optimal final receptor concentration per well across all tested GABA_A_ receptor isoforms. nanoBRET readings and analysis were carried out as with on-cell nanoBRET assays.

### Cell-based fluorescence assays

HEK293T cells (ATCC CRL-3216) were maintained in DMEM (Gibco) supplemented with 10% foetal bovine serum (Gibco), 1% L-gln (Sigma-Aldrich) and 1% non-essential amino acids (Fisher Scientific) at 37 °C and 5% CO₂. Cells were seeded onto 0.01% poly-L-lysine-coated black 96-well plates (Sigma-Aldrich; Greiner Bio-One) at ~45,000 cells/well and transfected ~16 h later with 200 ng GABA_A_ receptor subunit DNA per well using polyethyleneimine (PEI; Sigma-Aldrich). At 36-48 h post-transfection, cells were washed with PBS/0.5% BSA (Sigma-Aldrich) and stained for 20 minutes with either DyLight488-conjugated streptavidin (Strep488; Invitrogen, Cat. No. 21832; 1:200) or Fc-fused nanobodies (20–0.02 µM) followed by 5 minute incubation with Alexa Fluor 488-conjugated goat anti-human IgG secondary antibody (Thermo Fisher Scientific, Cat. No. A-11013; Lot No: 2247411; goat polyclonal; 1:500). After three PBS washes, fluorescence was measured using a CLARIOstar Plus microplate reader (BMG LABTECH; Ex 490 nm/Em 530 nm).

### Two-electrode voltage clamp oocyte electrophysiology

Electrophysiological properties of the GABA_A_ receptors were investigated in *Xenopus* oocytes using the automated two-electrode voltage clamp HiClamp system (Multichannel System, Germany). Electrodes were made out of borosilicate glass and filled with 3 M KCl and recordings performed using a ground agar bridge. Data were digitised at 100 Hz and filtered at 20 Hz and analysis was done using proprietary software developed in Matlab (Mathworks Inc.). Unless indicated cells were superfused during the entire experiments with OR2 containing in mM: NaCl 82.5, KCl 2.5, HEPES 5, CaCl_2_·2H_2_O 1.8, MgCl_2_ 1, pH 7.4. Expression of the desired GABA_A_ receptor was observed 3 to five days following injection with 15 nL of mRNA containing the desired subunit combination. Typically, batches of 96 oocytes were dispensed in microtiter plates and injected using the automated RoboInject device (Multichannel System, Germany). Dose response curves were fitted using Prism (version 10.4) “Dose-response – Stimulation, log(agonist) versus response (three parameters)” which uses equation Y=Bottom + (Top-Bottom)/(1 + 10^(LogEC50-X)^). GABA doses used for EC_1-3_ responses were determined based on identifying GABA doses versus the I_max_ that gave 1–3% of the maximal GABA response. This was performed for WT receptors and each mutated receptor. Each construct then received the appropriate fixed GABA dose across oocytes for generating the DS2-Me modulation curves.

### HEK cell patch clamp electrophysiology

Nanobody modulation was measured using HEK cell electrophysiology. One day prior to experiments, 8 ml of DMEM was pre-incubated for 10 min at room temperature with 96 μl lipofectamine2000 (Thermofisher) and 48 μg plasmid DNA, then added to a single T175 cm2 flask containing HEK293T cells (30–50 % confluency) and 2 ml DMEM (supplemented with 10 % foetal calf serum, L-Gln and non-essential amino acids). After 3 hrs this media was removed and replaced by DMEM supplemented with 10 % foetal calf serum. Plasmids were mixed in 1α:1β ratio supplemented with 3% plasmid encoding enhanced green fluorescent protein (EGFP) to assess transfection efficiency (typically 50–80 %). 18–24 h later cells were washed with PBS, incubated in 4 ml TrypLE (Gibco) for 7 min at 37 °C, suspended in 21 ml DMEM supplemented with 10 % foetal calf serum and L-Gln, centrifuged at 100 *g* for 1.5 min, then suspended in 50 ml Freestyle 293 Expression Medium (Gibco) and placed in a shaking incubator (130 rpm, 37 °C, 8 % CO_2_) for 30 min. 25 ml cell suspension was then centrifuged at 100 *g* for 1.5 min, and suspended in 4 ml external recording solution (mM): 137 NaCl, 4 KCl, 1 MgCl2, 1.8 CaCl2, 10 HEPES, and 10 D-Glucose, pH 7.4 ( ~ 305 mOsm). The internal recording solution contained (mM): 140 CsCl, 5 NaCl, 1 MgCl2, 10 HEPES, 5 EGTA, 0.2 ATP, pH 7.35 (» 295 mOsm). Electrophysiological recordings were performed at room temperature using an Ionflux16 (Molecular Devices) in ensemble mode, with series resistance compensation set at 80 % and cells held at −60 mV.

### Single channel recordings and analysis

Adherent HEK293T cells grown in DMEM supplemented with 10 % FBS and 100 U/mL pen/strep were seeded onto poly-D-lysine coated glass coverslips at least 1 hr prior to calcium/phosphate transfection with EGFP, empty vector (pRK5), wt α4, wt β3 and wt δ DNA clones in a ratio of 1:20:0.2:0.2:0.6–a total of 4 μg DNA solution was added to each coverslip. Single channel electrophysiology was performed 16–20 hrs after transfection.

Single channel currents from α4β3δ receptors, activated by 30 μM GABA, or 30 μM GABA + 1 μM DS2-Me, were recorded in outside-out patches voltage clamped at –70 mV using short shank borosilicate glass pipettes (6–7 MΩ; Harvard Apparatus GC150-7.5) with parafilm-coated tips. Single channel currents were recorded using an Axopatch 200B and filtered at 5 kHz (4-pole Bessel filter) before digitising at 20 kHz with a Digidata 1550B. The fixed time resolution of the system was set at 80 μs. WinEDR (v4.2.0) was used for post-record filtering ( ~ 1.5 kHz) and analysis of single channel parameters.

Single channel transitions between open and closed states were detected using a 50% threshold cursor. Transitions were then manually inspected and any unclear transitions were rejected to form an idealised record (usually 5000–10,000 transitions from each record), from which individual open and closed dwell times were measured, as well as open channel current amplitudes. Single channel current amplitude histograms were fitted with Gaussian components to define the mean current. Channel state frequency distribution histograms were constructed from the individual open and closed times and analysed by fitting a mixture of exponentials, defined by:

$$y\left(t\right)=\mathop{\sum }\limits_{i=1}^{n}\left({Ai}/\tau i\right)\exp (-t/\tau i)$$

where A_i_ is the area of the i^th^ component to the distribution and τ_i_ represents the corresponding exponential time constant. A Levenberg-Marquardt non-linear least-squares fitting routine was used to determine the values of individual exponential components.

The determination of a critical closed time (τ_crit_) to define bursts of GABA channel activity was performed between τ_2-closed_ and τ_3-closed_ as previously described^54^.

### Cell lines

HEK 293 T cells used for electrophysiology and cell staining were obtained from ATCC. Expi293F GnTI- cells used for protein production for Cryo-EM were obtained from Thermofisher. Further authentication of cell lines was not performed for this study. Mycoplasma testing was not performed for this study.

### Reporting summary

Further information on research design is available in the Nature Portfolio Reporting Summary linked to this article.

## Supplementary information

Supplementary Information

Reporting Summary

Transparent Peer Review file

## Source data

Source Data

## Data Availability

The authors declare that the data supporting the findings of this study are available within the paper. The source data underlying Figs. 1d, e, 3d, 4e–f, Supplementary Fig. 1a, b, 1d, e, 1h, 3a–c, 4, 5e, 5g, 6d, 6g–n are provided as a Source Data file. Atomic model coordinates for αβδ-Nb24-GABA, αβδ-Nb24-Mb30-GABA, and αβδ-Nb24-Mb30-GABA-DS2Me have been deposited in the Protein Data Bank with accession codes 9SLM, 9SL1, 9SLD, respectively. Cryo-EM density maps have been deposited in the Electron Microscopy Data Bank with accession codes EMD-55007, EMD-54981, EMD-54987 respectively. Previously published structural models used for comparative analysis in this study are GABA_A_ receptor α1β3γ2 bound by GABA and alprazolam (6HUO) and GABA_A_ receptor α1β3γ2 bound by GABA and bicuculline (6HUK). Source data are provided with this paper.
