# Supplementary material for: Molecular arrangement and modulation of extrasynaptic αβδ GABAA receptors
Source: Nat Commun. 2026 Aug 18;17:9889. doi: 10.1038/s41467-026-76879-5 (PMC13582853; doi:10.1038/s41467-026-76879-5)
Supplement: Supplementary file 1 [file 41467_2026_76879_MOESM1_ESM.pdf]

Supplementary Information for

Molecular arrangement and modulation of extrasynaptic αβδ $GABA_A$ receptors

Stephanie A. Nestorow[1], Wan-Na Chen[1], Daniel Bertrand[2], Ayla A. Wahid[1], Caroline E. Wyatt[1], Martin Mortensen[3], Thomas Clairfeuille[4], Michael Reutlinger[4], Gregoire Friz[5], Els Pardon[6,7], Jan Steyaert[6,7], Graham Ladds[1], Giuseppe Cecere[4], Trevor G. Smart[3], Maria-Clemencia Hernandez[5], Lisa Joedicke[4]*, Paul S. Miller[1]*

* e-mail: pm676@cam.ac.uk, lisa.joedicke@roche.com

The PDF file includes:

Supplementary Figures 1-7 and Table 1

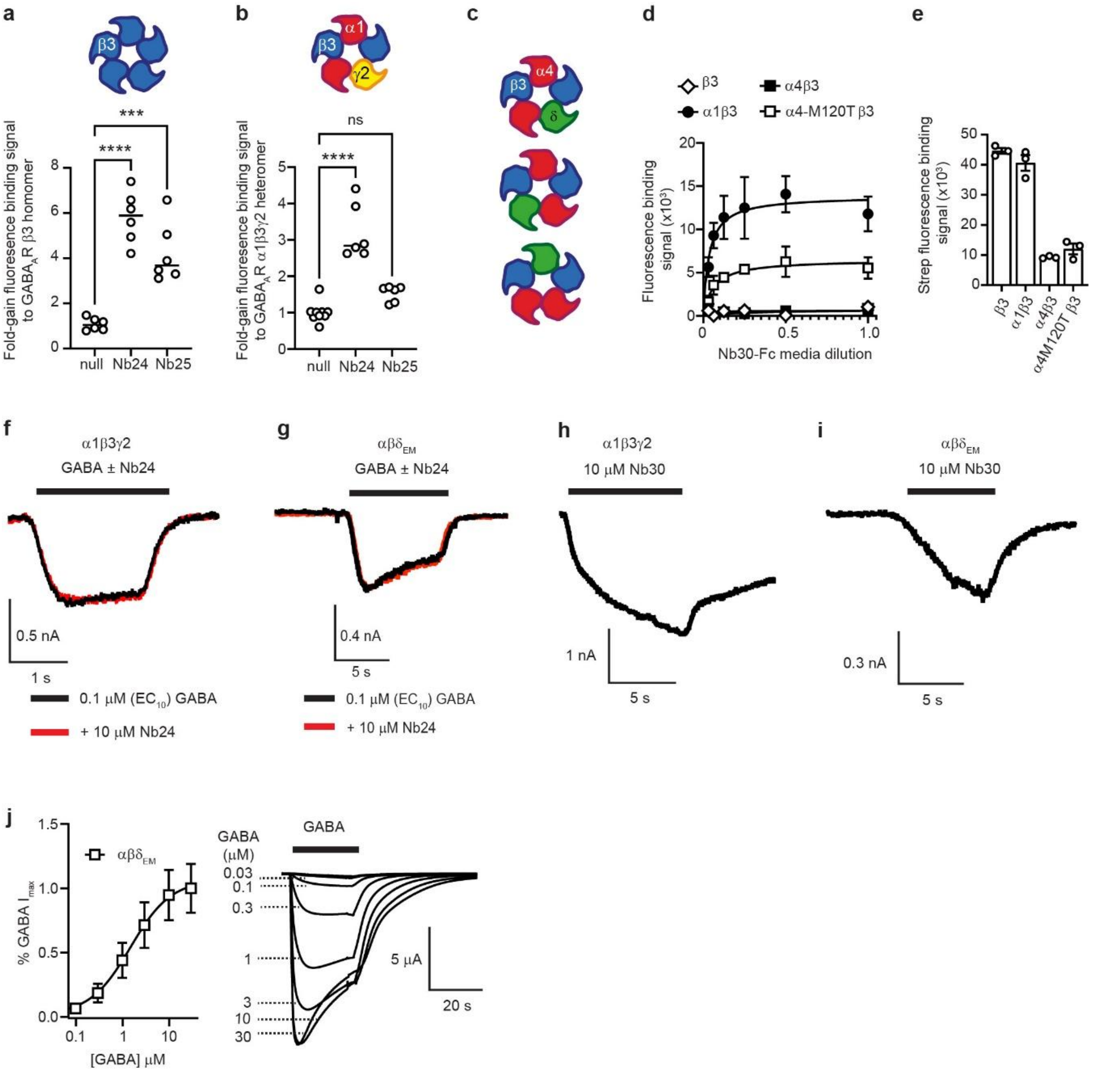

Supplementary Fig.1. Fiducial and receptor construct evaluation for Cryo-EM. a, Binding by two alternative nanobody-Fc proteins expressed in culture media to cells expressing β3 homomers, versus null signal from fluorescent secondary antibody-AF488 alone (null vs Nb24, $P < 0.0001$; null vs Nb25, $P = 0.0002$). **b,** Equivalent to **a,** but for α1β3γ2 receptors, showing significant binding only by Nb24, which binds receptors lacking a β-β interface (null vs Nb25, $P < 0.0001$; null vs Nb25, $P = 0.0766$). **c,** Schematic representation of three possible arrangements for αβδ receptors comprising a 2α:2β:1δ stoichiometry lacking a β-β interface. **d,** Cell-binding assay measuring binding by Nb30-Fc at different dilutions of the culture media it was expressed in showing a single α4-M120T mutation permits binding to α4 subunits in αβ heteromers, comparing β3 homomers, α1β3 (1:1), α4β3 (1:1) and α4-M120Tβ3 (1:1) **e,** Strep fluorescence binding signal to an SBP-tag fused to the N-terminus of each $GABA_AR$ confirming that differences in surface expression do not account for differences in Nb30 binding to difference subtypes. **f** and **g,** Whole-cell patch-clamp recording from an HEK293T cell expressing **f**, α1β3γ2 heteromers or **g**, $αβδ_{EM}$, showing no modulation by 10 μM purified Nb24 of an EC_{10} GABA response. **h** and **i,** Same as **f**, and **g,** but for Nb30 acting alone as an agonist. **j,** GABA dose response curve measured from two-electrode voltage clamp recordings for αβδ-EM and accompanying current responses from one oocyte. Data are mean ± s.e.m. For **a, b, d, e,** n = 6, 6, 3, 3, with each n being a separate binding experiment. For **j,** n = 5, with each n representing a single dose response curve from a

different oocyte. For **a**, and **b**, One-way ANOVA Dunnett's multiple comparisons; ns = $P > 0.05$; *** = $P < 0.001$; **** = $P < 0.0001$.

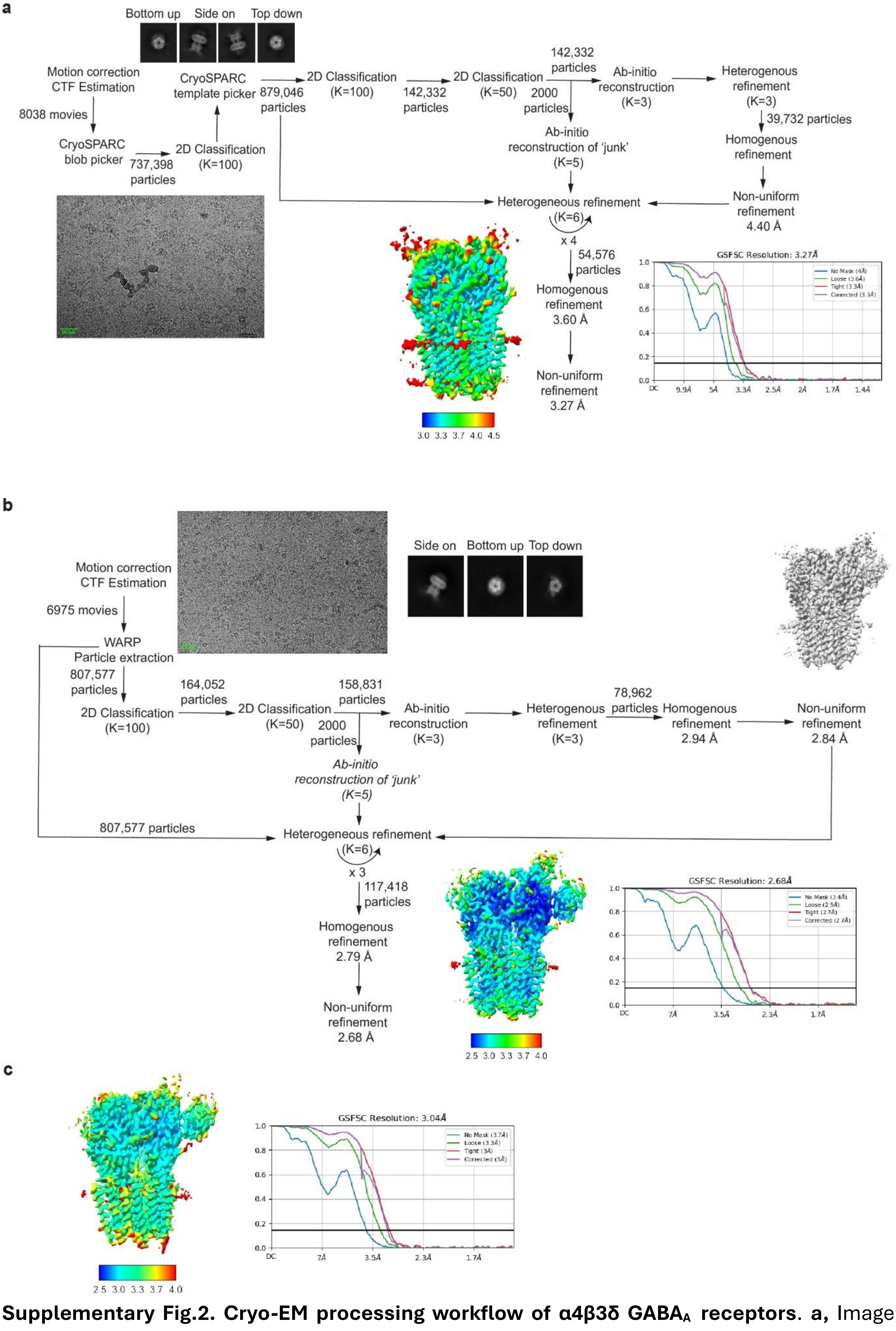

Supplementary Fig.2. Cryo-EM processing workflow of α4β3δ $GABA_A$ receptors. a, Image processing workflow for α4β3δ + GABA bound by Nb24 in cryoSPARC from micrograph processing

(Motion correction and CTF estimation) through 2D classification and 3D ab initio model generation. Refinement of the 'best' model which shows optimal particle distribution was used to generate a 'reference' map that becomes input for multiple rounds of heterogenous refinement alongside 'junk' classes using the original particle extraction ouput. The largest class is subject to further rounds of refinement with global and local map resolution shown, following data collection. **b,** Image processing workflow for α4β3δ + GABA bound by Nb24 and Mb30, following micrograph motion correction, CTF estimation and particle picking and extraction using WARP. Extracted particles were then further processed in cryoSPARC through 2D classes, 3D ab initio model to final model after iterative heterogenous refinement rounds as previously described. Global and local map resolution shown. **c,** α4β3δ + GABA + DS2-Me bound by Nb24 and Mb30, showing final map refinement and local map resolution, processing workflow as shown in **b**. Each structure was determined from a single sample preparation.

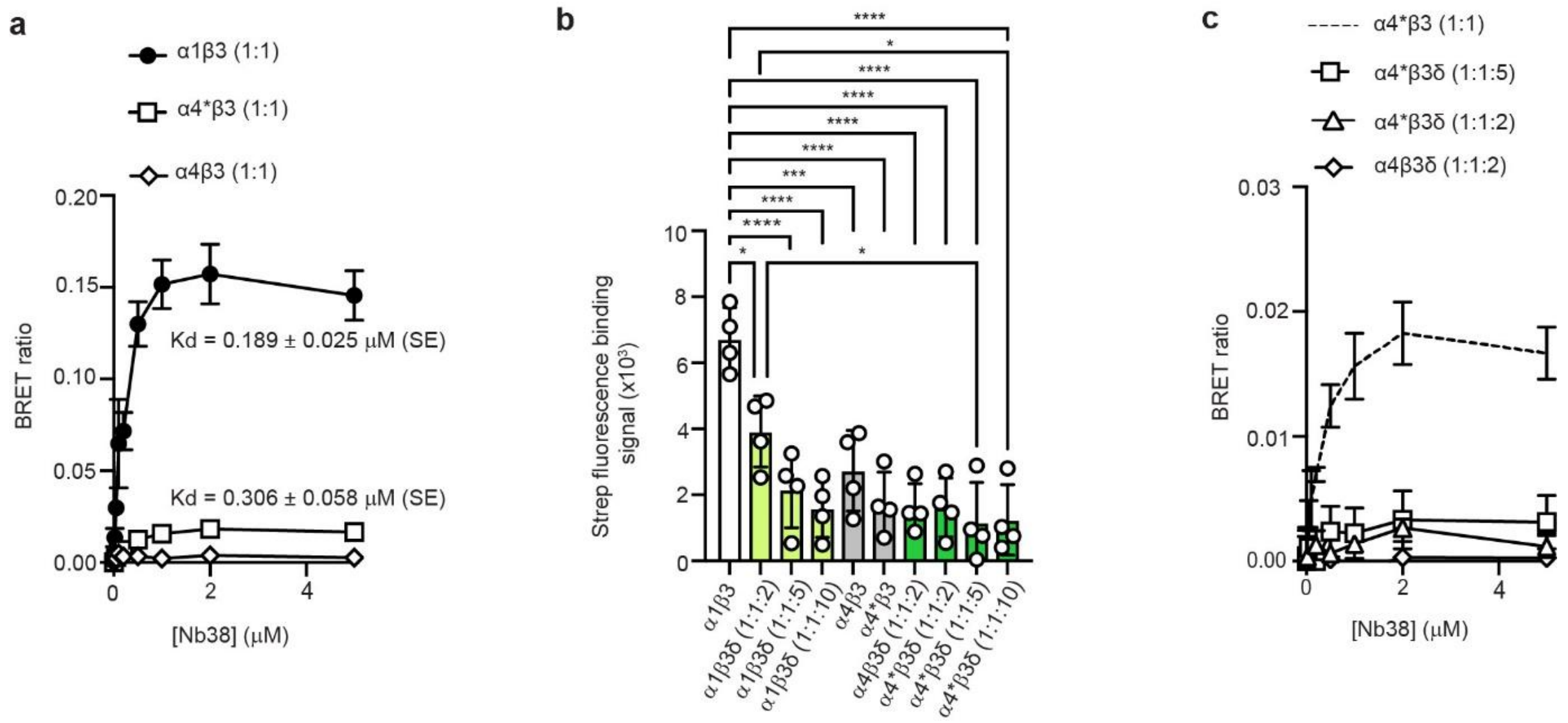

Supplementary Fig.3. Use of nanoBRET to assess α4β3δ $GABA_A$ receptor stoichiometry in cell membranes. **a,** On-cell nanoBRET Nb38 binding curves for αβ receptors, comparing binding to α1β3 and α4*β3 receptors with similar affinity but no binding to wild-type α4β3 receptors, which lack the necessary Nb38 binding residues. For α4*β3 the maximum nanoBRET fluorescence:bioluminesence ratio ($nanoBRET_{MAX}$) is reduced ~9-fold. This is presumably due to an ~60 % reduction in cell surface expression of α4*β3 versus α1β3 receptors as shown in **b**, which reduces the cell surface bioluminescent receptor binding pool versus the background intracellular bioluminescent receptor non-binding pool, meaning less fluorescent Nb38 binds relative to the bioluminescence signal so reducing the fluorescence:bioluminescence ratio and $nanoBRET_{MAX}$. **b**, Strep fluorescence binding signal to an SBP-tag fused to the N-terminus of the α-subunit showing that the α4 and α4* subunit exhibit reduced surface expression versus the α1 subunit and that α-subunit surface expression drops as the δ transfection ratio increases. α1β3 vs α1β3δ, *P* = 0.017; α1β3 vs α1β3δ, *P* < 0.0001; α1β3 vs α1β3δ, *P* < 0.0001; α1β3 vs α4*β3, *P* < 0.0001; α1β3 vs α4β3, *P* = 0.0002; α1β3 vs α4*β3δ, *P* < 0.0001; α1β3 vs α4*β3δ, *P* < 0.0001; α1β3 vs α4*β3δ, *P* < 0.0001; α1β3 vs α4β3δ, *P* < 0.0001; α1β3δ vs α4*β3δ, *P*= 0.0199; α1β3δ vs α4*β3δ, *P* = 0.0265. **c,** The low α4*β3 nanoBRET ratio (dashed line – original data plotted in **a,**) is only slightly above the reliable detection threshold (0.01). As was seen for the α1β3δ receptors (Fig. 1e) addition to αβ receptors of δ-subunits and saturation with high plasmid ratios (to ensure all pentamers include a δ-subunit) further reduces the on-cell assay $nanoBRET_{MAX}$ below the threshold for reliable confirmation of Nb38 binding (for α4*β3δ at 1:1:2 and 1:1:5 plasmid DNA). Data represent mean ± s.e.m., *n* = 3 for BRET and *n* = 4 for strep staining, for all subtypes tested, across separate experiments. K_d values in **a,** (mean ± s.e.m) were compared using an unpaired *t*-test; no significant difference was detected (*p* > 0.05). In **b**, statistics determined using a one-way ANOVA followed by Tukey's multiple comparisons test; *, P < 0.05; ****, P < 0.001.

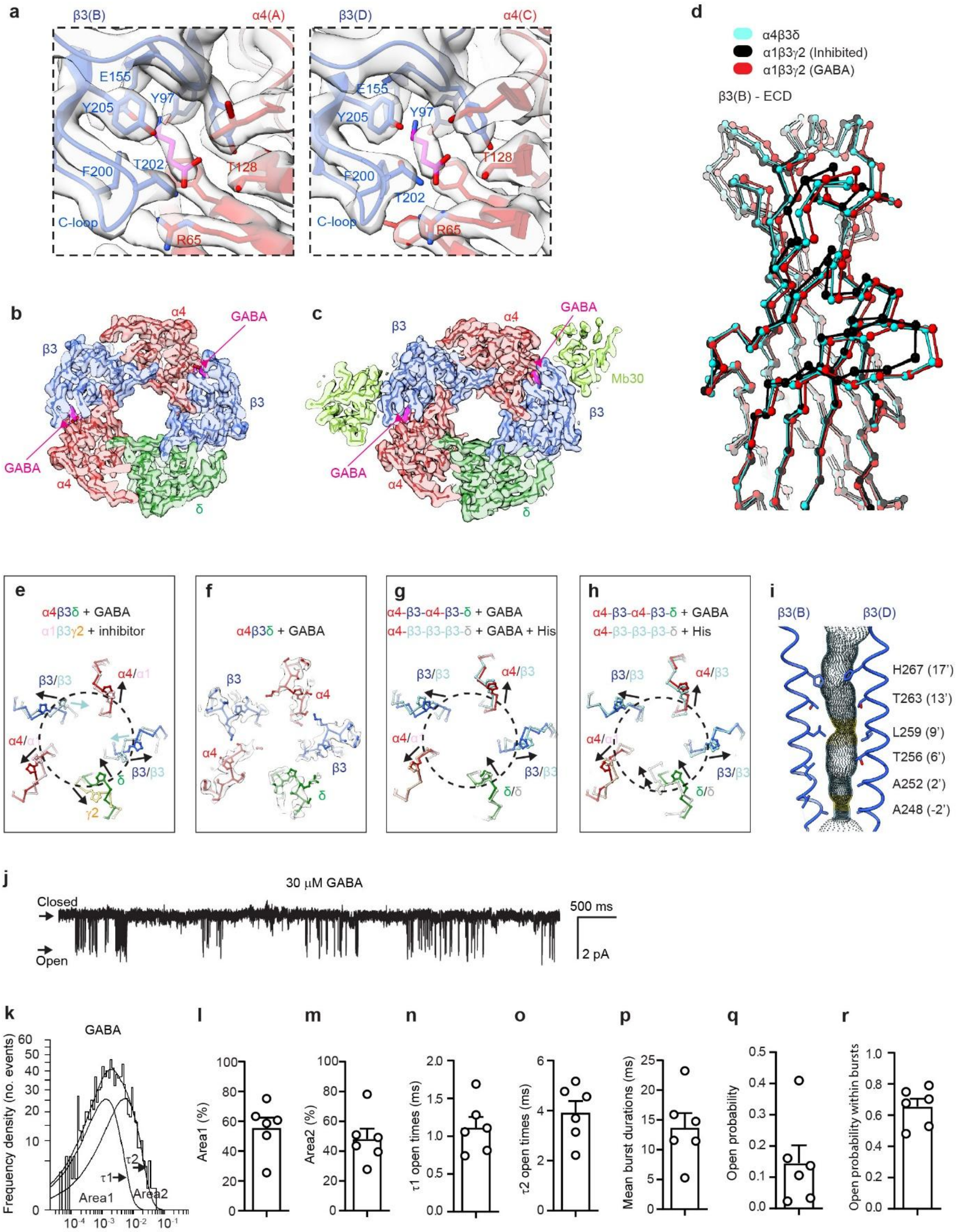

Supplementary Fig.4. α4β3δ $GABA_A$ receptor conformation in response to GABA. a, Ribbon representation showing side chains and GABA ligand as sticks (GABA – magenta) in both agonist β/α ECD pockets (Chains B/A and D/C. Map density shown in grey. **b**, and **c,** Cryo-EM map slice views through the α4β3δ receptor ECD showing GABA density (magenta) present in both GABA pockets (indicated by pink arrows), in the absence, **b,** or presence **c,** of Mb30. **d,** Global structural alignment of β-subunit ECD (chain B) of α4β3δ-GABA (Nb24+Mb30)(cyan) showing it is tilted to assume the α1β3γ2-GABA (PDB:6HUO – red) conformation rather than the α1β3γ2-bicuculline (inhibited; 6HUK – black) conformation. **e,** Top-down view of the M2-M3 loops shown in Cα

representation with conserved proline side chain shown as a positional marker, comparing α4β3δ-GABA with α1β3γ2-bicuculline (inhibited; PDB:6HUK – lighter shades). Both β-subunit M2-M3 loops are positioned outwards for α4β3δ-GABA, compared to inwards for α1β3γ2-bicuculline (light blue arrows). **f,** α4β3δ receptor M2-M3 loop model build and cryo-EM map density (grey). **g, h,** Same as **e,** but comparisons are versus receptors containing three β-subunits, **g,** α4-β3-β3-β3-δ-GABA-His-His (His – histamine; PDB:7QN7) and **h,** α4-β3-β3-β3-δ-His-His-His (PDB:7QN9). **i,** Side-on-view of the permeation pathway. Only the β3 subunits are shown, for clarity. B3 pore lining residues are labelled. Pore narrowings are shown by yellow dots, at the 9' and -2' gates. At the 9' gate the β3 subunit adjacent to δ (δ not shown) positions L259 pointing into the pore, whereas the β3 subunit non-adjacent to δ is rotated away from the pore, creating an asymmetric narrowing. **j,** Outside-out patch single channel recording from HEK293T cell showing channel openings of recombinant α4β3δ $GABA_A$ receptor(s) induced by 30 μM GABA. **k-r,** Single channel open time histogram from α4β3δ-patch activated with 30 μM GABA showing two open time populations (τ1-2). The similar sized areas under the exponentials, **k,** are summarised from *n* = 6 patches/cells in **l,** (Area1; 56 ± 6.8 %) and **m,** (Area2; 48 ± 6.9 %), and the respective open time constants are shown in **n,** (τ1; 1.1 ± 0.14 ms), and **o,** (τ2; 3.9 ± 0.46 ms), **p,** Mean burst durations (14 ± 2.4 ms), **q,** the open probability (0.14 ± 0.058), and **r,** the open probability within bursts (0.66 ± 0.051) are also shown. Value bars are means ± s.e.m., *n* = 6.

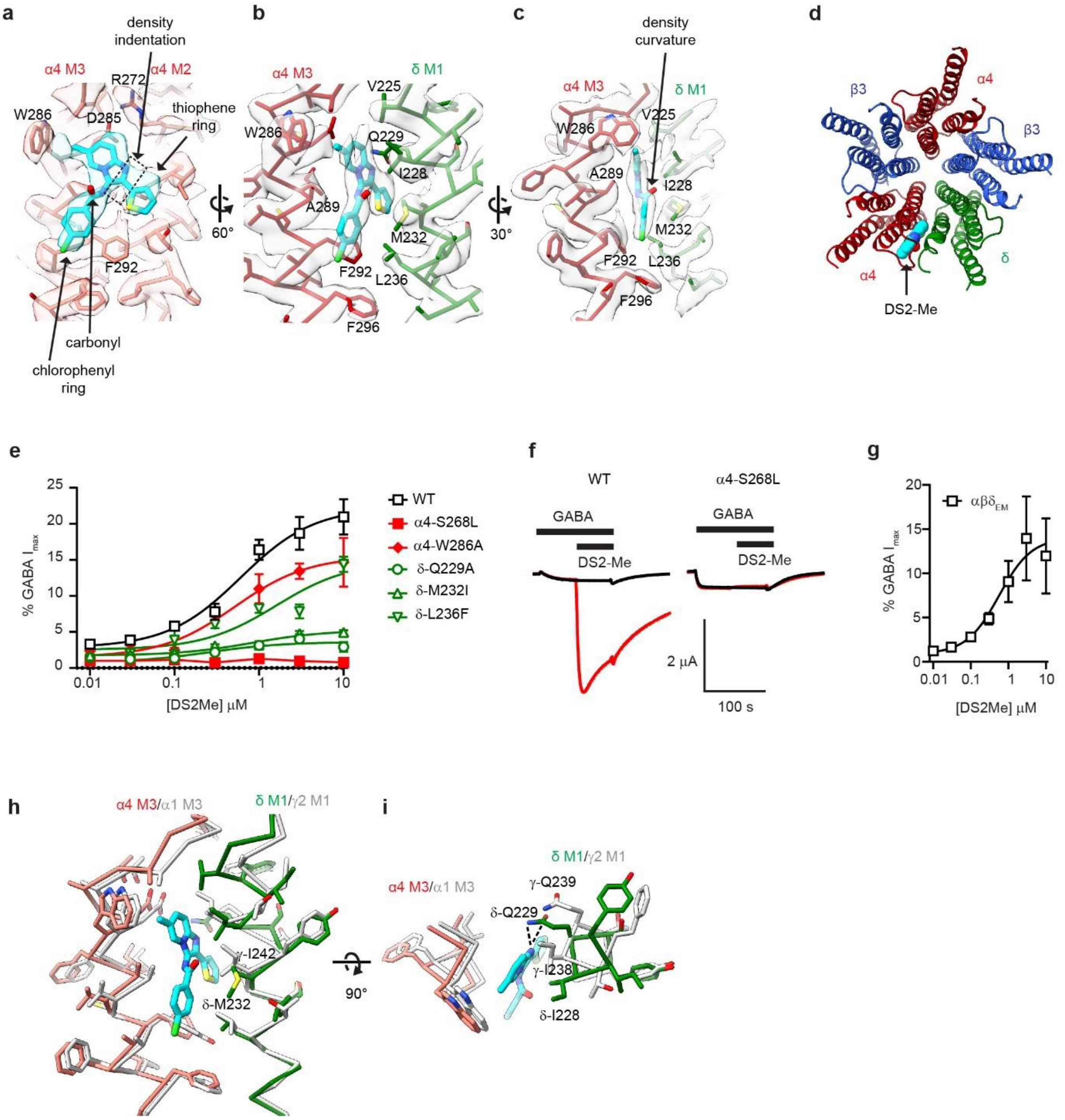

Supplementary Fig.5. DS2-Me binds at the α-δ interface. a, b, c, Three side-on viewing angles of the DS2-Me structural model pocket built into the cryo-EM map (grey). In **a,** an indentation in the ligand density is indicated (dashed box), which places the thiophene ring facing the pore. In **c,** a curvature in the density is indicated (black arrow) which was used to orient the carbonyl group connecting to the end phenyl group. **d,** Top-down ribbon representation of the α4β3δ TMD helices showing DS2-Me in sphere representation (cyan) bound at an α4-δ interface pocket. **e,** DS2-Me dose response curves measured from two-electrode voltage clamp recordings for α4β3δ receptors, wild-type versus ligand pocket mutations. **f,** Current responses from one oocyte each for wild-type and α4-S268L receptors stimulated by an EC_{1-3} GABA dose alone (black traces) or an EC_{1-3} GABA dose and subsequent co-application with 1 μM Ds2-Me (red traces). **g,** DS2-Me dose response curve for αβδ-EM . **h**, Side-on view of a C_α and side chain stick representation of a global structural alignment of α4β3δ and α1β3γ2 receptors, highlighting the single residue difference in the DS2-Me pocket, δ M232 versus γ2 I242. **i,** Equivalent top-down view highlighting the top of the pocket where the position of the M1 helix diverges between δ and γ2, causing the pocket lining γ2 I238 to sterically clash with DS2-Me, and Q239 to lose favourable putative polar

binding interactions with the imidazole moiety. Data are mean ± s.e.m. For **e,** and **g,** each data point represents the mean of 3-6 data points, with each recording of a DS2-Me concentration application being to a different oocyte.

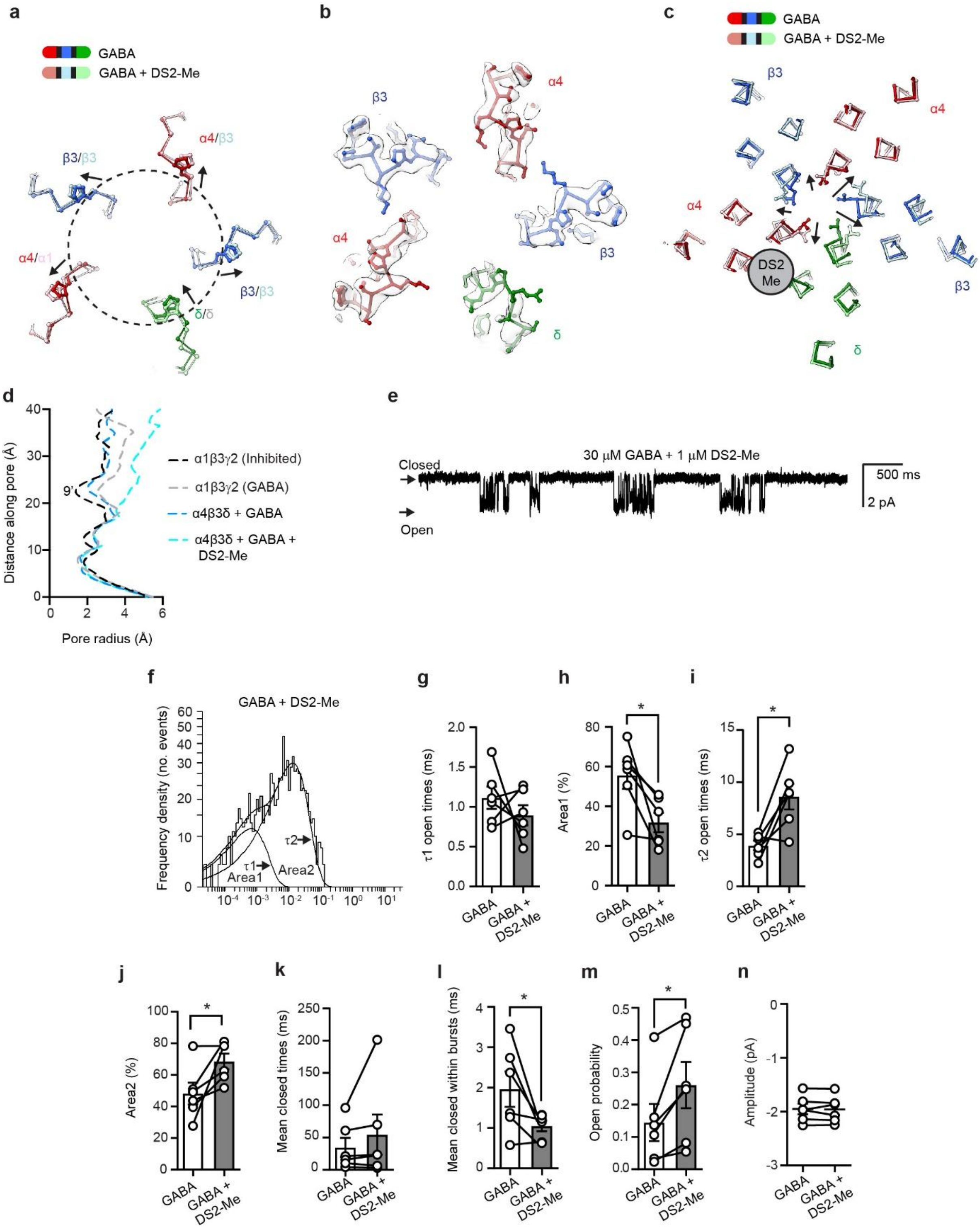

Supplementary Fig.6. α4β3δ GABA$_A$ receptor conformation in response to DS2-Me. a, Top-down view of the M2-M3 loops shown in Cα representation with conserved proline side chain shown as a positional marker, comparing α4β3δ-GABA with α4β3δ-GABA+DS2-Me, showing the same pentameric conformation. **b,** α4β3δ-GABA-DS2-Me structural M2-M3 loop model build and cryo-EM map density (grey). **c,** Cross-section of the α4β3δ-GABA-DS2-Me pore at the 9' Leu ring in C$_α$ representation with leucine side chains shown, revealing the changes in positions induced by DS2-Me binding (black arrows), such that the 9' Leu residues are retracted to enlarge the pore at the 9' gate. d, Pore radius plot of the α4β3δ-GABA receptor in the presence versus absence of DS2-Me, and compared to the α1β3γ2-GABA receptor (PDB:6HUO) and α1β3γ2-bicuculline

receptor (inhibited, PDB:6HUK). The 9’ gate is indicated. **e,** Outside-out patch single channel recording from HEK293T cell showing channel openings of recombinant α4β3δ $GABA_A$ receptor(s) induced by 30 μM GABA and 1 μM DS2-Me. **f,** Single channel open time histogram from α4β3δ activated with 30 μM GABA + 1 μM DS2-Me showing two open time populations (τ1-2). Compared with data from only GABA activation (white bars), when adding DS2-Me (grey bars), the τ1 open duration, **g,** was unchanged ($1.1 \pm 0.14_{G}$ ms versus $0.89 \pm 0.13_{G+DS2\text{-}Me}$ ms; p = 0.42), whereas Area1, **h,** decreased (from $56 \pm 6.8_{G}$ % to $32 \pm 4.9_{G+DS2\text{-}Me}$ %; p = 0.02); however, the τ2 open duration, **i,** increased (from $3.9 \pm 0.46_{G}$ to $8.6 \pm 1.2_{G+DS2\text{-}Me}$ ms; p = 0.015), and Area2, **j,** also increased (from $48 \pm 6.9_{G}$ to $68 \pm 5.0_{G+DS2\text{-}Me}$ %; p = 0.039). Overall mean closed times, **k,** are unaltered ($35 \pm 15_{G}$ ms vs. $54 \pm 31_{G+DS2\text{-}Me}$ ms; p = 0.30) whist mean closed durations within bursts decreased (**l,** $2.0 \pm 0.44_{G}$ ms vs. $1.1 \pm 0.13_{G+DS2\text{-}Me}$ ms; p = 0.046). The overall open probability, **m,** increased (from $0.14 \pm 0.058_{G}$ to $0.26 \pm 0.076_{G+DS2\text{-}Me}$ ms; p = 0.033), whereas no change in single channel current amplitude, **n,** was observed (-1.95 ± 0.099 pA vs. -1.96 ± 0.10 pA; p = 0.78). All statistical comparisons utilised paired t-tests, * = $P < 0.05$. Value bars are means ± s.e.m., $n = 6$.

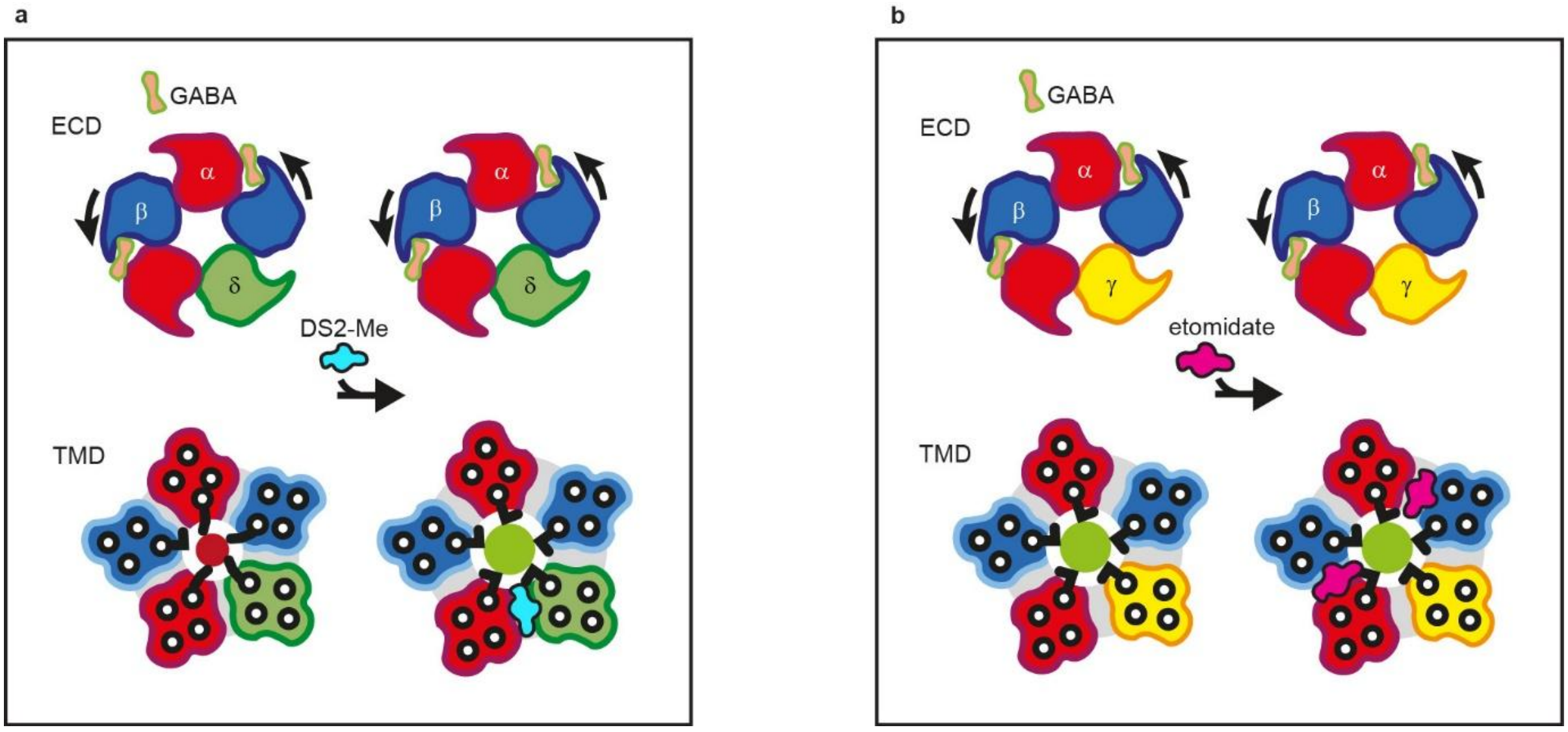

Supplementary Fig.7. Schematic comparing DS2-Me δ-selective α4β3δ PAM modulation versus etomidate non-selective αβγ PAM modulation. Top-down views of the ECD (upper) and TMD (lower) of, **a,** an αβδ receptor, and, **b,** an αβγ receptor. TMD helices = white circles. Closed pore = red circle; open pore = green circle; pore-facing 9' residue = subtle bent frond; rotated-out 9' residue = L-shaped frond. In **a,** for αβδ receptors GABA binding stabilises tilting of each β-subunit ECD but only one 9' residue rotates out of the pore (left). Upon DS2-Me binding all 9' residues rotate outwards and the pore opens. In **b,** for αβγ receptors GABA binding has the same effect on the ECD, however for the TMD all five 9' residues have rotated outwards to open the pore. Etomidate binding continues to stabilise an already open pore.

Supplementary Table 1 | Cryo-EM data collection, refinement and validation statistics.

**Data Collection**	α4^{M120T}β3δ + GABA + DS2-Me (Mb30 + Nb24)	α4^{M120T}β3δ + GABA (Mb30 + Nb24)	α4^{M120T}β3δ + GABA (Nb24)
PDB Code	PDB-9SLD	PDB-9SL1	PDB-9SLM
EMDB Code	EMDB-54987	EMDB-54981	EMDB-55007
Facility	Biochemistry Cryo-EM Facility, University of Cambridge	Biochemistry Cryo-EM Facility, University of Cambridge	Electron Bio-Imaging Centre (EBIC) UK
Microscope	Titan Krios,	Titan Krios,	Titan Krios,
Magnification	165k	165k	130k
Voltage (kV)	300	300	300
Exposure Time (s)	4.39	4.39	1.44
Number of Frames	150	150	107
Electron Exposure (e^-/Å)	53.60	51.12	49.95
Detector	F4i counting	F4i counting	Gatan K3
Pixel Size (Å/pixel)	0.729	0.729	0.645
Micrographs	7912	6975	8038
**Reconstruction**			
Initial Number of Particles	689,954	807,577	1,606,604
Final Number of Particles	110,542	117,418	47,269
Symmetry Imposed	C1	C1	C1
Box Size (pixels)	480	480	384
Map Resolution (Å)	3.04	2.68	3.27
FSC Threshold	0.143	0.143	0.143
Map Sharpening B Factor (A^2)	74.2	54.8	89.5
**Refinement**			
Number of Non-H Atoms	17,510	17,541	15,621
Protein Residues	2,152	2,160	1,907
N-Glycans	15	15	15
Ligands	3	2	2
Water	2	2	2
MolProbity Score	1.59	1.43	1.23
Clashscore	6.15	4.79	4.08
Poor Rotamers (%)	0.05	0.05	0.00
Bond Length (Å) RMSD	0.005	0.007	0.004
Bond Angle (°C) RMSD	0.439	0.482	0.536
Ramachandran Favoured (%)	96.28	96.89	97.83
Ramachandran Outliers (%)	0.00	0.00	0.00
