# Supplementary material for: Molecular arrangement and modulation of extrasynaptic αβδ GABAA receptors
Source: Nat Commun. 2026 Aug 18;17:9889. doi: 10.1038/s41467-026-76879-5 (PMC13582853; doi:10.1038/s41467-026-76879-5)
Supplement: Supplementary file 2 [file 41467_2026_76879_MOESM2_ESM.pdf]

nature portfolio

Corresponding author(s): Paul S. Miller
Last updated by author(s): Jul 6, 2026

Reporting Summary

Nature Portfolio wishes to improve the reproducibility of the work that we publish. This form provides structure for consistency and transparency in reporting. For further information on Nature Portfolio policies, see our Editorial Policies and the Editorial Policy Checklist.

Statistics

For all statistical analyses, confirm that the following items are present in the figure legend, table legend, main text, or Methods section.

n/a	Confirmed	
☐	☒	The exact sample size (n) for each experimental group/condition, given as a discrete number and unit of measurement
☐	☒	A statement on whether measurements were taken from distinct samples or whether the same sample was measured repeatedly
☐	☒	The statistical test(s) used AND whether they are one- or two-sided<br>*Only common tests should be described solely by name; describe more complex techniques in the Methods section.*
☒	☐	A description of all covariates tested
☐	☒	A description of any assumptions or corrections, such as tests of normality and adjustment for multiple comparisons
☐	☒	A full description of the statistical parameters including central tendency (e.g. means) or other basic estimates (e.g. regression coefficient) AND variation (e.g. standard deviation) or associated estimates of uncertainty (e.g. confidence intervals)
☐	☒	For null hypothesis testing, the test statistic (e.g. F, t, r) with confidence intervals, effect sizes, degrees of freedom and P value noted<br>*Give P values as exact values whenever suitable.*
☒	☐	For Bayesian analysis, information on the choice of priors and Markov chain Monte Carlo settings
☒	☐	For hierarchical and complex designs, identification of the appropriate level for tests and full reporting of outcomes
☒	☐	Estimates of effect sizes (e.g. Cohen's d, Pearson's r), indicating how they were calculated

Our web collection on statistics for biologists contains articles on many of the points above.

Software and code

Policy information about availability of computer code

Data collection	Cryo-EM datasets were collected using EPU software (Thermo Fisher Scientific, versions 3.7–3.9). Electrophysiological recordings were acquired using the IonFlux16 platform (Molecular Devices), and single-channel recordings were performed using an Axopatch 200B amplifier. Small-molecule ligand geometry restraint files were generated using the Grade Web Server (Global Phasing). Initial nanobody models were generated using AlphaFold2. No custom code was used for data collection.
Data analysis	Cryo-EM data were processed using Warp (v1.0.9) and cryoSPARC (v4.4.1–4.7.1). Model building and refinement were performed using Coot (v0.9.8.8), HOLE, Phenix (v1.21.1), Molprobity and ChimeraX (v1.6.1). Two-electrode voltage clamp oocyte electrophysiology data were digitized at 100 Hz and filtered at 20 Hz and analysis was done using proprietary software developed in Matlab (Mathworks Inc.).WinEDR (v4.2.0) was used for post-recording filtering and analysis of single-channel parameters. Statistical analyses were performed using GraphPad Prism (v10.4). No custom code was used for data analysis.

For manuscripts utilizing custom algorithms or software that are central to the research but not yet described in published literature, software must be made available to editors and reviewers. We strongly encourage code deposition in a community repository (e.g. GitHub). See the Nature Portfolio guidelines for submitting code & software for further information.

Data

Policy information about availability of data

All manuscripts must include a data availability statement. This statement should provide the following information, where applicable:

- Accession codes, unique identifiers, or web links for publicly available datasets
- A description of any restrictions on data availability
- For clinical datasets or third party data, please ensure that the statement adheres to our policy

The data supporting the findings of this study are available within the paper. The source data underlying Figures 1d-e, 3d, 4e-f, Supplementary Figure 1a-b, 1d-e, 1h, 3a-c, 4, 5e, 5g, 6d, 6g-n are provided as a Source Data file. Atomic models were built using model PDB-7QN7 from the Protein Data Bank. Atomic model coordinates for αβδ-Nb24-GABA, αβδ-Nb24-Mb30-GABA, and αβδ-Nb24-Mb30-GABA-DS2Me have been deposited in the Protein Data Bank with accession codes 9SLM [http://doi.org/10.2210/pdb9SLM/pdb], 9SL1 [http://doi.org/10.2210/pdb9SL1/pdb], 9SLD [http://doi.org/10.2210/pdb9SLD/pdb], respectively. Cryo-EM density maps have been deposited in the Electron Microscopy Data Bank with accession codes EMD-55007 [https://www.ebi.ac.uk/pdbe/entry/emdb/EMD-55007], EMD-54981 [https://www.ebi.ac.uk/pdbe/entry/emdb/EMD-54981], EMD-54987 [https://www.ebi.ac.uk/pdbe/entry/emdb/EMD-54987] respectively. Previously published structural models used for comparative analysis in this study are GABAA receptor α1β3γ2 bound by GABA and alprazolam 6HUO [http://doi.org/10.2210/pdb6HUO/pdb] and GABAA receptor α1β3γ2 bound by GABA and alprazolam [http://doi.org/10.2210/pdb6HUK/pdb]. There are no restrictions on data availability.

Research involving human participants, their data, or biological material

Policy information about studies with human participants or human data. See also policy information about sex, gender (identity/presentation), and sexual orientation and race, ethnicity and racism.

Reporting on sex and gender	Not relevant to this study because no human participants, human-derived material, or human data were used.
Reporting on race, ethnicity, or other socially relevant groupings	Not relevant to this study because no human participants or human-derived datasets were included.
Population characteristics	No human participants were involved in this study.
Recruitment	No human participants were recruited for this study.
Ethics oversight	Ethics approval was not required because this study did not involve human participants, human-derived material, or human data.

Note that full information on the approval of the study protocol must also be provided in the manuscript.

Field-specific reporting

Please select the one below that is the best fit for your research. If you are not sure, read the appropriate sections before making your selection.

☒ Life sciences ☐ Behavioural & social sciences ☐ Ecological, evolutionary & environmental sciences

For a reference copy of the document with all sections, see nature.com/documents/nr-reporting-summary-flat.pdf

Life sciences study design

All studies must disclose on these points even when the disclosure is negative.

Sample size	Sample sizes were not predetermined using statistical methods. Cryo-EM datasets were collected so that sufficient particle numbers were obtained to produce high-resolution reconstructions that met accepted quality criteria for structural interpretation and model validation. Electrophysiological and biochemical experiments were performed with sample sizes consistent with established practice in the field and sufficient to demonstrate reproducible findings across independent experiments. Exact sample sizes (n) are reported in the relevant figure legends.
Data exclusions	Following standard cryoSPARC and processing pathways, best representative 2D and 3D classes were selected for the final particle reconstructions. Poor quality and irrelevant other classes were discarded. No other data exclusions apply.
Replication	Electrophysiological and biochemical experiments were independently repeated ≥3 times using independent biological replicates, yielding similar results. Protein structures were consistent across repeated purifications. For each single structure two independent maps of each cryo-EM sample were generated in order to estimate resolution according to the recommended procedures in the field (the 'gold standard'). No findings failed to reproduce.
Randomization	Randomisation was not relevant to this study because samples were not allocated into experimental groups.
Blinding	Blinding was not relevant to this study because data collection and analysis were not subject to investigator-dependent group allocation or outcome assessment.

Reporting for specific materials, systems and methods

We require information from authors about some types of materials, experimental systems and methods used in many studies. Here, indicate whether each material, system or method listed is relevant to your study. If you are not sure if a list item applies to your research, read the appropriate section before selecting a response.

Materials & experimental systems

n/a	Involved in the study
☐	☒ Antibodies
☐	☒ Eukaryotic cell lines
☒	☐ Palaeontology and archaeology
☒	☐ Animals and other organisms
☒	☐ Clinical data
☒	☐ Dual use research of concern
☒	☐ Plants

Methods

n/a	Involved in the study
☒	☐ ChIP-seq
☒	☐ Flow cytometry
☒	☐ MRI-based neuroimaging

Antibodies

Antibodies used: Goat anti-Human IgG (H+L) Cross-Adsorbed Secondary Antibody, Alexa Fluor™ 488 (Invitrogen, Thermo Fisher Scientific; Cat. No. A-11013; Lot No: 2247411; goat polyclonal) was used for fluorescence-based cell staining at a dilution of 1:500. Rho-1D4 antibody was purchased from the University of British Columbia (https://ubc.flintbox.com/technologies/0f1ef64b-fa5d-4a58-9003-3e01f6f672a6). 50 mg of 1D4 antibody in 20 ml phosphate buffered saline was coupled to 3.3 g dry powdered cyanogen bromide beads (see methods). The megabody MbF3 was obtained from the laboratory of Professor Jan Steyeart (VUB).

Validation: A-11013 is a commercially available reagent validated by the manufacturer for immunofluorescence applications and was used according to the manufacturer's instructions. The Rho-1D4 antibody was previously validated to bind the sequence TETSQVAPA (PMID: 6188482). We have previously validated it can be used to affinity purify GABA-A receptors with the TETSQVAPA C-terminal tag (PMID: 24909990). The megabody MbF3 was characterised and published elsewhere (PMID: 33408403).

Eukaryotic cell lines

Policy information about cell lines and Sex and Gender in Research

Cell line source(s): HEK293T cells (ATCC, Cat. No. CRL-3216; female) were used for electrophysiology and fluorescence-based cell staining experiments. Expi293F cells (Gibco, Thermo Fisher Scientific, Cat. No. A14527; female) were used for recombinant expression of soluble proteins. Expi293F GnTI^{-} cells (Gibco, Thermo Fisher Scientific, Cat. No. A39240; female) were used for recombinant protein expression for cryo-EM studies.

Authentication: Further authentication of cell lines was not performed for this study.

Mycoplasma contamination: Mycoplasma testing was not performed for this study.

Commonly misidentified lines (See ICLAC register): HEK293-derived cell lines used in this study are not listed in the ICLAC database of commonly misidentified cell lines.

Plants

Seed stocks: *Report on the source of all seed stocks or other plant material used. If applicable, state the seed stock centre and catalogue number. If plant specimens were collected from the field, describe the collection location, date and sampling procedures.*

Novel plant genotypes: *Describe the methods by which all novel plant genotypes were produced. This includes those generated by transgenic approaches, gene editing, chemical/radiation-based mutagenesis and hybridization. For transgenic lines, describe the transformation method, the number of independent lines analyzed and the generation upon which experiments were performed. For gene-edited lines, describe the editor used, the endogenous sequence targeted for editing, the targeting guide RNA sequence (if applicable) and how the editor was applied.*

Authentication: *Describe any authentication procedures for each seed stock used or novel genotype generated. Describe any experiments used to assess the effect of a mutation and, where applicable, how potential secondary effects (e.g. second site T-DNA insertions, mosiacism, off-target gene editing) were examined.*
