# Supplementary material for: Molecular arrangement and modulation of extrasynaptic αβδ GABAA receptors
Source: Nat Commun. 2026 Aug 18;17:9889. doi: 10.1038/s41467-026-76879-5 (PMC13582853; doi:10.1038/s41467-026-76879-5)
Supplement: Supplementary file 3 [file 41467_2026_76879_MOESM3_ESM.pdf]

nature portfolio

Peer Review File

Molecular arrangement and modulation of extrasynaptic αβδ $GABA_A$ receptors

Corresponding Author: Dr Paul Miller

This file contains all reviewer reports in order by version, followed by all author rebuttals in order by version.

Version 0:

Reviewer comments:

Reviewer #1

(Remarks to the Author)
This is a very nice and elaborate study that reveals the binding site of DS2-Me in an a4b3d receptor with two GABA sites. Cryo-EM, mutational analysis and ElPhys were combined to generate a very convincing data package, which is presented well including the discussion of limitations. I have no critical remarks and congratulate all contributing authors,
margot ernst

Reviewer #2

(Remarks to the Author)
This manuscript by Nestorow and colleagues is a methodologically sound study using cryo-EM, nanoBRET, and electrophysiology coupled with site-directed mutagenesis to: 1) describe the subunit stoichiometry and structure of α4β3δ GABA-A receptors; 2) describe a structural basis for GABA partial agonism; and 3) identify a functional DS2 binding in the interface between α4 and δ subunits in the transmembrane domain site responsible for its PAM effect. These data represent a significant addition to our understanding of the structure and pharmacological modulation of δ-containing extra synaptic GABA-A receptors. The identification of novel nanobodies and megabodies for fiducial identification of GABA-A subunits is an additional contribution of the paper. The data supporting these points is well presented and convincing.
An important point that is not resolved in the paper is the discrepancy between the receptor stoichiometries for α4β3δ receptors previously identified by Sente et al (αβββδ, βδ; ref 6) and the exclusive αβαβδ stoichiometry observed in the current study. This discrepancy is attributed to expression using transient vs stable transfection and the use of different ratios of subunit DNA for transfection; however, both studies tried using different subunit ratios for transfection. The conclusion offered is that all of the αβδ subunit stoichiometries are plausible. The stark discrepancy between these two studies is perplexing. It seems that using the Nb24 nanobody as a fiducial should have identified any pentamers with alternative stoichiometries. It also seems that the analytical pipeline used in the Sente paper should have identified particles (at least at the level of 2-D classification) that lacked β/β interfaces. Additional discussion and possibly experimentation to address this point would enhance the manuscript.
DS2 is known to be a more efficacious and more potent PAM in α4βδ than in α1βδ receptors. Since structures of both receptors were obtained in this study, it would be useful to discuss whether differences between the α1/δ and α4/δ interfaces can explain this difference.

Reviewer #3

(Remarks to the Author)
This manuscript addresses an important question in δ-containing GABAA receptor structural pharmacology by combining fiducial-assisted cryo-EM with nanoBRET and electrophysiology. The work provides structural evidence for an α-β-α-β-δ arrangement with two orthosteric GABA pockets and proposes a δ-selective transmembrane pocket for DS2-Me at the α-δ interface. By integrating structural and functional data, the study identifies a δ-selective transmembrane modulatory site, providing a framework for understanding GABA efficacy in αβδ receptors and for guiding subtype-selective modulator design. However, several points require clarification or additional support to ensure that the conclusions are stated with appropriate precision.

1. Please standardize the subunit nomenclature throughout the manuscript, as the current usage (e.g., "α4β3δ", "β3-β3", "α1β3γ2") is confused in the text and figure legends. I suggest all numbers be formatted as subscripts. For clarity, "α4M120T" should preferably be written as "α4-M120T". The designation "α4M120Tβ3δEM" is difficult for readers to interpret quickly. I recommend using the simplified notation "αβδ-EM" instead.

2. There appears to be an inconsistency in fiducial/megabody naming across the manuscript. The main text refers to Nb30/Mb30, whereas the Methods list Nb130/Mb130 (Methods, line 422 "Fiducials included 5 μM Nb24, 5 μM Nb130, 2.6 μM Mb130, 3 μM MbF3"), and the same Mb130 notation also appears in Extended Data Fig. 2 (line 671) and Extended Data Table 1. Fiducial identity directly affects interpretation, such as epitope specificity, occupancy, functional and conformational effects. Please verify the correct fiducials used for each dataset and standardize the nomenclature throughout the manuscript.

3. Extended Data Fig. 1e (N-terminal SBP tag/Strep-based surface readout) is potentially an important control, but its current framing makes it difficult to assess which specific comparisons it is intended to support. In the manuscript, surface expression is required both to interpret Nb30-Fc binding differences across receptor constructs/subtypes and to rationalize the reduced on-cell nanoBRETMAX. To strengthen this point and avoid over-extension, please (i) explicitly state which receptor pairs/conditions are being compared in Fig. 1d, Extended Data Fig. 1d, and Extended Data Fig. 3. (ii) provide the SBP/Strep surface signals under those same transfection ratios/conditions, and (iii) if possible, present Nb30-Fc binding and nanoBRETMAX normalized to the SBP/Strep surface signal.

4. Line 112 "Fig. 1f" appears to refer to a panel that is not clearly identifiable. Please verify all figure references so readers can unambiguously locate the supporting data.

5. The α4-M120T substitution is introduced to enable Nb30 binding, and Nb30 acts as an agonist. In Extended Data Fig. 1f, g, the functional profiling of Nb24 and Nb30 is performed in whole-cell recordings on α1β3γ2 heteromers. Given that these controls were obtained on a different receptor subtype, please consider providing a minimal matched control on the α4M120Tβ3δEM construct (or α4β3δ) under ±Nb24/Nb30. This would help to confirm that baseline GABA-evoked currents (e.g., peak amplitude/Imax and a simple kinetic metric) are not substantially altered.

6. The very low global Cα RMSD between structures ±Mb30 supports overall similarity. Because functionally critical differences can be highly localized, the manuscript would benefit from a brief local analysis of key gating elements (9′ Leu ring, -2′ ring, δ M2-M3 loop, β M2-M3 loops), for example via local RMSD or per-residue displacement. In addition, a short statement summarizing whether 3D classification revealed minor alternative conformations even at low abundance would help readers interpret the structural ensemble.

7. The inward/outward assignment of the β-subunit M2-M3 loop appears inconsistent between the Extended Data Fig. 4e legend (line 720 "β-subunit M2-M3 loops are positioned inwards for α4β3δ-GABA, compared to outwards for α1β3γ2-bicuculline") and the main text (lines 145-147). Please verify the directionality assignments and harmonize the wording across the manuscript to avoid confusion regarding ECD-TMD coupling.

8. Fig. 3d compares DS2-Me potentiation across multiple α4/δ pocket mutants using EC1-3 GABA responses. For clarity and reproducibility, please specify how the EC1-3 GABA concentration was determined: was EC1-3 calibrated for each mutant construct or each oocyte based on its own GABA concentration-response curve and Imax definition, or was a fixed GABA concentration used for all constructs? In addition, the text refers to "EC1-2" (line 198) whereas the Fig. 3d legend indicates "EC1-3" (line 220). Please clarify this definition.

9. While the cryo-EM density places DS2-Me at the α-δ transmembrane (TMD) interface in the present α4β3δ construct, prior DS2 studies (notably in α4β1δ receptors) have proposed additional candidate sites, including an α4(+)δ(–) extracellular-domain (ECD) interface and two intersubunit TMD pockets at α4-β1 interfaces (α4(+)β1(–) and α4(–)β1(+)). Moreover, structure-activity relationship analyses within the imidazopyridine series indicate that 5-position substitutions can substantially affect potency, selectivity and overall pharmacological profile. We therefore suggest clarifying whether the proposed TMD site at the α-δ interface is expected to apply to DS2 or is specific to DS2-Me. A brief discussion of plausible reasons for differences from earlier DS2 models would also be helpful.

10. The pocket-mutant panel convincingly supports the α/δ TMD site for DS2-Me potentiation. However, δ-selectivity is currently supported mainly by the δ M232I loss-of-function test based on the γ2-equivalent residue. If feasible, a complementary gain-of-function swap at the γ2-equivalent position would help establish a more direct causal link to DS2-Me selectivity; otherwise, a brief note on this limitation would be helpful.

11. The text line 209 refers to an "Ile-Asn residue pair", whereas the Extended Data 5i labels the corresponding residues as γ2 I238 and γ2 Q239. Please check the residue identities across the main text and Extended Data to avoid confusion.

12. The manuscript proposes an R272-centred interaction network linking DS2-Me binding to M2 movement and 9′ gate opening. If feasible, testing an α4 R272 mutant to assess whether DS2-Me potentiation is reduced would strengthen this mechanistic model. If such mutants are not functional, a brief note on feasibility/limitations would help frame the interpretation.

13. The Conclusion (lines 265 and 266) states that nanoBRET is used to "demonstrate" an α-β-α-β-δ arrangement for α1β3δ.

Because Nb38-nanoBRET primarily reports the presence of a β(+)/α(–) interface, it does not by itself distinguish among alternative permutations within a 2α:2β:1δ stoichiometry. I suggest adjusting the wording to “supports/consistent with” and explicitly distinguishing the cryo-EM fiducial-based arrangement assignment for α4β3δ from the interface-presence evidence for α1β3δ.

Version 1:

Reviewer comments:

Reviewer #2

(Remarks to the Author)
Thank you for your thoughtful responses to my questions. I enjoyed reading and reviewing your work.

Reviewer #3

(Remarks to the Author)
The authors have addressed all my raised comments, and I therefore recommend acceptance of the manuscript.

Open Access This Peer Review File is licensed under a Creative Commons Attribution 4.0 International License, which permits use, sharing, adaptation, distribution and reproduction in any medium or format, as long as you give appropriate credit to the original author(s) and the source, provide a link to the Creative Commons license, and indicate if changes were made.
In cases where reviewers are anonymous, credit should be given to 'Anonymous Referee' and the source.
The images or other third party material in this Peer Review File are included in the article’s Creative Commons license, unless indicated otherwise in a credit line to the material. If material is not included in the article’s Creative Commons license and your intended use is not permitted by statutory regulation or exceeds the permitted use, you will need to obtain permission directly from the copyright holder.
To view a copy of this license, visit https://creativecommons.org/licenses/by/4.0/

REVIEWER COMMENTS

Reviewer #1 (Remarks to the Author):

This is a very nice and elaborate study that reveals the binding site of DS2-Me in an a4b3d receptor with two GABA sites. Cryo-EM, mutational analysis and ElPhys were combined to generate a very convincing data package, which is presented well including the discussion of limitations. I have no critical remarks and congratulate all contributing authors,
margot ernst
Dear Margot, thank you so much for taking the time to review our manuscript and provide this positive feedback. It is much appreciated and we appreciate the congratulations. Cheers, Paul.

Reviewer #2 (Remarks to the Author):

This manuscript by Nestorow and colleagues is a methodologically sound study using cryo-EM, nanoBRET, and electrophysiology coupled with site-directed mutagenesis to: 1) describe the subunit stoichiometry and structure of α4β3δ GABA-A receptors; 2) describe a structural basis for GABA partial agonism; and 3) identify a functional DS2 binding in the interface between α4 and δ subunits in the transmembrane domain site responsible for its PAM effect. These data represent a significant addition to our understanding of the structure and pharmacological modulation of δ-containing extra synaptic GABA-A receptors. The identification of novel nanobodies and megabodies for fiducial identification of GABA-A subunits is an additional contribution of the paper. The data supporting these points is well presented and convincing.

We are very grateful to Reviewer #2 for the positive feedback and for taking the time to review our study.

An important point that is not resolved in the paper is the discrepancy between the receptor stoichiometries for α4β3δ receptors previously identified by Sente et al (αβββδ, βδ; ref 6) and the exclusive αβαβδ stoichiometry observed in the current study. This discrepancy is attributed to expression using transient vs stable transfection and the use of different ratios of subunit DNA for transfection; however, both studies tried using different subunit ratios for transfection. The conclusion offered is that all of the αβδ subunit stoichiometries are plausible. The stark discrepancy between these two studies is perplexing. It seems that using the Nb24 nanobody as a fiducial should have identified any pentamers with alternative stoichiometries. It also seems that the analytical pipeline used in the Sente paper should have identified particles (at least at the level of 2-D classification) that lacked β/β interfaces. Additional discussion and possibly experimentation to address this point would enhance the manuscript.

We thank reviewer #2 for this comment. We now provide an expanded discussion of this discrepancy in the conclusion (lines 293 to 332). In our case transient transfection allows control of the DNA ratios and because we included slightly more α4-subunit DNA

than β3-DNA (1.25:1) this might have precluded observing receptors with β-β interfaces, in contrast to those observed in the previous study. In their study, the use of stable cell lines, even with trying to increase the amount of α4 DNA is unlikely to directly translate into more α4 subunits due to the low copy number of insertion into the genomic DNA, and so the relative expression profile of each subunit becomes stochastic. This is discussed more thoroughly in the new added section.

DS2 is known to be a more efficacious and more potent PAM in α4βδ than in α1βδ receptors. Since structures of both receptors were obtained in this study, it would be useful to discuss whether differences between the α1/δ and α4/δ interfaces can explain this difference.

To be clear, we did not solve structures of α1βδ receptors in this study. We performed some nanoBRET work on α1β3δ receptors to investigate the stoichiometry, but we did not solve a structure. Therefore, we would prefer not to discuss a potential molecular explanation without an α1β3δ structure for comparison because there might be differences in arrangement and/or conformational responsiveness to agonists, etc, that will complicate any extrapolation. We have clarified in the discussion that cryo-EM structures are specific to the α4β3δ isoform, and that nanoBRET was used to examine inclusion of the α(+)/β(–) interface into both α1β3δ and α4β3δ receptors isoforms – lines 286-288.

Reviewer #3 (Remarks to the Author):

This manuscript addresses an important question in δ-containing GABAA receptor structural pharmacology by combining fiducial-assisted cryo-EM with nanoBRET and electrophysiology. The work provides structural evidence for an α-β-α-β-δ arrangement with two orthosteric GABA pockets and proposes a δ-selective transmembrane pocket for DS2-Me at the α-δ interface. By integrating structural and functional data, the study identifies a δ-selective transmembrane modulatory site, providing a framework for understanding GABA efficacy in αβδ receptors and for guiding subtype-selective modulator design. However, several points require clarification or additional support to ensure that the conclusions are stated with appropriate precision.

We are very grateful to reviewer #3 for taking the time to read this manuscript and provide feedback. Responses are further below.

1. Please standardize the subunit nomenclature throughout the manuscript, as the current usage (e.g., “α4β3δ”, “β3-β3”, “α1β3γ2”) is confused in the text and figure legends. I suggest all numbers be formatted as subscripts. For clarity, “α4M120T” should preferably be written as “α4-M120T”. The designation “α4M120Tβ3δEM” is difficult for readers to interpret quickly. I recommend using the simplified notation “αβδ-EM” instead.

- We note that receptor subtype nomenclature in the $GABA_A$ receptor field is commonly formatted without subscripts (e.g. α4β3δ, α1β2γ2), and we have

therefore retained this convention throughout the manuscript for consistency with prior literature.

- Changed a4^{M120T} to a4-M120T throughout
- Also, we now add "cryo-EM construct, α4^{M120T}β3$δ_{EM}$ (hereafter termed αβδ-EM)", lines 76-77.

2. There appears to be an inconsistency in fiducial/megabody naming across the manuscript. The main text refers to Nb30/Mb30, whereas the Methods list Nb130/Mb130 (Methods, line 422 "Fiducials included 5 μM Nb24, 5 μM Nb130, 2.6 μM Mb130, 3 μM MbF3"), and the same Mb130 notation also appears in Extended Data Fig. 2 (line 671) and Extended Data Table 1. Fiducial identity directly affects interpretation, such as epitope specificity, occupancy, functional and conformational effects. Please verify the correct fiducials used for each dataset and standardize the nomenclature throughout the manuscript.

- Nanobody nomenclature standardised throughout manuscript text and figures (i.e. Mb130 → Mb30 and Nb130→Nb30)

3. Extended Data Fig. 1e (N-terminal SBP tag/Strep-based surface readout) is potentially an important control, but its current framing makes it difficult to assess which specific comparisons it is intended to support. In the manuscript, surface expression is required both to interpret Nb30-Fc binding differences across receptor constructs/subtypes and to rationalize the reduced on-cell nanoBRETMAX. To strengthen this point and avoid over-extension, please (i) explicitly state which receptor pairs/conditions are being compared in Fig. 1d, Extended Data Fig. 1d, and Extended Data Fig. 3. (ii) provide the SBP/Strep surface signals under those same transfection ratios/conditions, and (iii) if possible, present Nb30-Fc binding and nanoBRETMAX normalized to the SBP/Strep surface signal.

- Regarding (i) the main text has been rewritten more precisely to allow easier comparison between the datasets. Lines 110-124. Of note, we now do not refer to EDF1d because to address point (ii) we have performed a new larger screen in parallel, which we now refer to instead.
- Regarding (ii) We now provide an in parallel comparison of strep surface expression for all the constructs tested by nanoBRET. This data is added as an extra panel in EDF3, new panel b. This allows for direct consideration of the strep surface receptor expression levels versus the $BRET_{max}$ values.
- Regarding (iii), the Nb30-Fc binding assay was only to validate that this Nb can be used as a fiducial in cryo-EM. It is not relevant to the nanoBRET study which was performed using Nb38 (which we never tested by Fc binding), so it would not be useful to compare the Nb30-Fc data to the nanoBRET data. Also, it's not possible to normalise the nanoBRETmax to the strep fluorescence because for some constructs, e.g. the α4β3δ ones both signals are very low and close to background (see new EDF3b vs 3a and 3c) and so the results are noisy and a ratio would not be meaningful. However, as we now provide new panel 3b alongside 3a and 3c this does now allow comparison from within the same figure to see how reducing surface expression (strep staining) drives down BRETmax.

4. Line 112 "Fig. 1f" appears to refer to a panel that is not clearly identifiable. Please verify all figure references so readers can unambiguously locate the supporting data.

- Line 124 - Full-length α4*β3δ receptors did reveal clear binding by Nb38. Reference changed to Fig. 1e. Fig. 1f written in error.

5. The α4-M120T substitution is introduced to enable Nb30 binding, and Nb30 acts as an agonist. In Extended Data Fig. 1f, g, the functional profiling of Nb24 and Nb30 is performed in whole-cell recordings on α1β3γ2 heteromers. Given that these controls were obtained on a different receptor subtype, please consider providing a minimal matched control on the α4M120Tβ3δEM construct (or α4β3δ) under ±Nb24/Nb30. This would help to confirm that baseline GABA-evoked currents (e.g., peak amplitude/Imax and a simple kinetic metric) are not substantially altered.

- Line 76 – We now provide matched responses also for α4M120Tβ3δEM receptors in panels EDF1g and i. "Whole cell patch-clamp recordings revealed that Nb24 does not modulate function, whilst Nb30 acts as an agonist for both α1β3γ2 receptors and the cryo-EM construct, $\alpha4^{M120T}\beta3\delta_{EM}$ (hereafter termed αβδ-EM) (Extended Data Fig. 1f-i)."

6. The very low global Cα RMSD between structures ±Mb30 supports overall similarity. Because functionally critical differences can be highly localized, the manuscript would benefit from a brief local analysis of key gating elements (9' Leu ring, -2' ring, δ M2-M3 loop, β M2-M3 loops), for example via local RMSD or per-residue displacement. In addition, a short statement summarizing whether 3D classification revealed minor alternative conformations even at low abundance would help readers interpret the structural ensemble.

- We now modify the statement "Comparison of the structural models of αβδ-EM bound by GABA, Nb24 and Mb30 versus without Mb30 reveal identical conformations throughout (C_α RMSD 0.12Å)" to "Comparison of the structural models of αβδ-EM bound by GABA, Nb24 and Mb30 versus without Mb30 reveal identical conformations throughout (overall C_α RMSD 0.12Å; 9' Leu ring, -2' ring, δ M2-M3 loop, β M2-M3 loops all < 0.15Å). Lines 149-151.
- Lines 94-96: Added additional comment regarding outcomes of additional classification: "No alternative subunit arrangements or alternative conformations were detected during follow-up 3D classification. The primary variability observed between classes was limited to differences in local resolution and map quality within the TMD."

7. The inward/outward assignment of the β-subunit M2-M3 loop appears inconsistent between the Extended Data Fig. 4e legend (line 720 "β-subunit M2-M3 loops are positioned inwards for α4β3δ-GABA, compared to outwards for α1β3γ2-bicuculline") and the main text (lines 145-147). Please verify the directionality assignments and harmonize the wording across the manuscript to avoid confusion regarding ECD-TMD coupling.

- The EDF4e figure legend has been corrected to be in line with the main text. Specifically, the sentence has been corrected from "β-subunit M2-M3 loops are positioned inwards for α4β3δ-GABA, compared to outwards for α1β3γ2-bicuculline" to "β-subunit M2-M3 loops are positioned **out**wards for α4β3δ-GABA, compared to **in**wards for α1β3γ2-bicuculline". This is consistent with the main text: "Each β-subunit M2-M3 loop, which sits under the ECD at the top of the TMD helical bundle, is positioned outwards, matching the α1β3γ2-GABA conformation (Fig. 2b) rather than inward as in the αβγ-inhibitor structure (Extended Data Fig. 4e,f)." We confirm the manuscript is now consistent throughout.

8. Fig. 3d compares DS2-Me potentiation across multiple α4/δ pocket mutants using EC1-3 GABA responses. For clarity and reproducibility, please specify how the EC1-3 GABA concentration was determined: was EC1-3 calibrated for each mutant construct or each oocyte based on its own GABA concentration-response curve and Imax definition, or was a fixed GABA concentration used for all constructs? In addition, the text refers to "EC1-2" (line 198) whereas the Fig. 3d legend indicates "EC1-3" (line 220). Please clarify this definition.

- Line 212 – main text now changed to $EC_{1\text{-}3}$ to be consistent with the figure legend.
- Lines 608-611. We now clarify the determination of $EC_{1\text{-}3}$ in the methods: "GABA doses used for $EC_{1\text{-}3}$ responses were determined based on identifying GABA doses versus the I_{max} that gave 1-3% of the maximal GABA response. This was performed for WT receptors and each mutated receptor. Each construct then received the appropriate fixed GABA dose across oocytes for generating the DS2-Me modulation curves."

9. While the cryo-EM density places DS2-Me at the α-δ transmembrane (TMD) interface in the present α4β3δ construct, prior DS2 studies (notably in α4β1δ receptors) have proposed additional candidate sites, including an α4(+)δ(–) extracellular-domain (ECD) interface and two intersubunit TMD pockets at α4-β1 interfaces (α4(+)β1(–) and α4(–)β1(+)). Moreover, structure-activity relationship analyses within the imidazopyridine series indicate that 5-position substitutions can substantially affect potency, selectivity and overall pharmacological profile. We therefore suggest clarifying whether the proposed TMD site at the α-δ interface is expected to apply to DS2 or is specific to DS2-Me. A brief discussion of plausible reasons for differences from earlier DS2 models would also be helpful.

- We have now included consideration of this in the discussion: "Two previous studies on the analogue DS2, which differs from DS2-Me only by the loss of a single methyl group, have each proposed their own alternative binding sites: one being between the α4 and δ-subunit ECDs at a homologous pocket to the neurotransmitter site; the other binding in the TMD at the α4–β1 interface. In the case of the proposed ECD pocket this was based on computational docking using a homology model from the α1β3γ2 subtype (PDB:6D6T), which differs from experimental δ-containing structures. Additionally, the authors

acknowledged that their putative binding site proposal could not explain the structure-activity relationship (SAR) around the thiophene substituent on the imidazo-pyridine core of DS2 or DS2-Me. Arguably, our structure provides evidence for the SAR on position 2 of the imidazo-pyridine core. This is based on the size of a small lipophilic pocket where the thiophene resides, alongside structural requirements for ligand geometry (specifically, the full planarity of the imidazo-pyridine core and the thiophene ring) that are not fulfilled when larger rings, such as a phenyl group, replace the thiophene. In the case of the proposed α4-β1 TMD pocket, the data was based on two mutations, α4(S303L) and β1(I289Q), which markedly reduced DS2 modulation. We show here that S303 is also present in our α4-δ TMD pocket (mature numbering S268), and we also observed that a leucine mutation ablates DS2-Me modulation (Fig. 3d). Given that S268 is important for both DS2 and DS2-Me modulation (shown in previous study and this study respectively) we propose both ligands bind the same α4-δ pocket determined here using cryo-EM, and that the β-subunit mutation might exert an indirect effect on modulation. Of note, our newly identified pocket fully explains the SAR around 5-position of imidazo-pyridine ligands, as well as the increased potency of DS2-Me compared to DS2. This is based on an additional interaction of the methyl residue at the 5-position with α4-W286. This finding is further supported by the drop in potency recorded for DS2-Me in the α4-W286A mutant (Fig 3d)." Lines 338-362.

10. The pocket-mutant panel convincingly supports the α/δ TMD site for DS2-Me potentiation. However, δ-selectivity is currently supported mainly by the δ M232I loss-of-function test based on the γ2-equivalent residue. If feasible, a complementary gain-of-function swap at the γ2-equivalent position would help establish a more direct causal link to DS2-Me selectivity; otherwise, a brief note on this limitation would be helpful.

- We have not tested this because direct residue swapping between δ and γ2 subunits is complicated by sizeable differences in local transmembrane helix geometry and side-chain trajectory at this interface. As a result, the γ2-equivalent position does not occupy an identical structural environment, and the simple reciprocal substitution suggested is not expected to recover binding due to other additional differences such as an upper helix Ile-Gln pair (present in both the γ2 and δ-subunit) being in a different position in the γ2-subunit which is predicted to clash with DS2-Me. This is noted in the main text "As well as the M232 residue difference, the upper γ2 M1 helix is rotated further than for δ, which changes the position of the Ile-Gln residue pair, causing the γ2 Ile to sterically clash with DS2-Me, and the γ2 Gln to lose favourable contacts (Extended Data Fig. 5i). Together, these differences likely explain the mode of DS2-Me δ-selectivity". Lines 224-226.

11. The text line 209 refers to an "Ile-Asn residue pair", whereas the Extended Data 5i labels the corresponding residues as γ2 I238 and γ2 Q239. Please check the residue identities across the main text and Extended Data to avoid confusion.

- Checked sequence, I238, Q239 is correct. Adjusted in line 224 to represent actual sequence and match figure i.e. Ile-Gln residue pair.

12. The manuscript proposes an R272-centred interaction network linking DS2-Me binding to M2 movement and 9' gate opening. If feasible, testing an α4 R272 mutant to assess whether DS2-Me potentiation is reduced would strengthen this mechanistic model. If such mutants are not functional, a brief note on feasibility/limitations would help frame the interpretation.

- We have performed testing of two mutations, however, the receptors have substantially reduced GABA Imaxes. This prevents performing DS2-Me modulation of $EC_{1\text{-}3}$ GABA responses and prevents straightforward interpretation of effects given the apparently important role of this residue in general protein function. We have added text in this regard to lines 248 to 254, as follows: “Substitution of R272 with alanine to remove charge and bulk or glutamine to remove charge but retain some polarity and bulk, both resulted in substantial, > 90 %, reductions in GABA I_{max} (100 μM) from 148 ± 43 nA to 8 ± 2 nA and 13 ± 2 nA respectively (n = 3), presumably due to an impact on gating, assembly or protein folding. These small responses from receptors containing the R272 mutations precluded measurement of DS2-Me modulation of GABA $EC_{1\text{-}3}$ responses and prevent direct confirmation of any role it might have in the DS2-Me PAM effect due to its apparent importance in general protein function.”

13. The Conclusion (lines 265 and 266) states that nanoBRET is used to “demonstrate” an α-β-α-β-δ arrangement for α1β3δ. Because Nb38-nanoBRET primarily reports the presence of a β(+)/α(–) interface, it does not by itself distinguish among alternative permutations within a 2α:2β:1δ stoichiometry. I suggest adjusting the wording to “supports/consistent with” and explicitly distinguishing the cryo-EM fiducial-based arrangement assignment for α4β3δ from the interface-presence evidence for α1β3δ.

- Lines 287-290, text edited to address that Nb38 specifically binds to reveal presence of an α(+)/β(–) interface rather than assuming specific arrangement of full pentamer – “We solve α4β3δ receptor structures revealing an α-β-α-β-δ arrangement. Separately, we also use nanoBRET nanosensors to support the presence of an α(+)/β(-) interface in full-length versions of both α1β3δ and α4β3δ receptor isoforms, which are absent from the previously determined α-β-β-β-δ subunit arrangements but will exist in our α-β-α-β-δ subunit arrangement.”
